# Phytochemical Analysis and In Vivo Anticancer Effect of *Becium grandiflorum*: Isolation and Characterization of a Promising Cytotoxic Diterpene

**DOI:** 10.3390/nu17071164

**Published:** 2025-03-27

**Authors:** Christeen Fahim, Maha R. A. Abdollah, Rola M. Labib, Nehal Ibrahim, Noha Swilam

**Affiliations:** 1 Department of Pharmacognosy, Faculty of Pharmacy, The British University in Egypt (BUE), El-Sherouk City 11837, Egypt; christeen.fahim@bue.edu.eg; 2 Department of Pharmacology and Biochemistry, Faculty of Pharmacy, The British University in Egypt (BUE), El-Sherouk City 11837, Egypt; maha.abdollah@bue.edu.eg; 3 Department of Pharmacognosy, Faculty of Pharmacy, Ain Shams University, Cairo 11566, Egypt; rolamilad@pharma.asu.edu.eg (R.M.L.); nehal.sabry@pharma.asu.edu.eg (N.I.)

**Keywords:** *Becium grandiflorum*, 18-epoxy-pimara-8(14),15-diene, diterpene, cytotoxicity, doxorubicin, Ehrlich ascites carcinoma

## Abstract

**Background:***Becium grandiflorum* is a fragrant perennial shrub of the Lamiaceae family. **Objectives**: The current study aimed to explore the cytotoxic potential of the *n*-hexane fraction from *Becium grandiflorum* aerial parts and, further, isolate its major diterpene and conduct in vitro and in vivo anticancer activities along with its molecular mechanism and synergy with doxorubicin. **Methods:** The hydroalcoholic extract of *Becium grandiflorum* aerial parts was fractionated, and the *n*-hexane fraction was analyzed via GC-MS. The major isolated diterpene, 18-epoxy-pimara-8(14),15-diene (epoxy-pimaradiene), was quantified using UPLC-PDA. Cytotoxicity assays were conducted on HCT-116, MCF-7, MDA-MB-231, and HepG2 cell lines. The synergistic effect with doxorubicin was tested on HepG2 cells. In vivo anticancer activity was evaluated using the Ehrlich ascites carcinoma model, and molecular docking analyzed Bax-Bcl2 interactions. **Results**: The *n*-hexane fraction contained 21 compounds, mainly oxygenated diterpenes, and the major isolated compound was epoxy-pimaradiene, with a quantity of 0.3027 mg/mg. *N*-Hexane fraction and epoxy-pimaradiene exhibited strong cytotoxicity against HepG2 cells, induced apoptosis, and G2/M arrest. The combination of epoxy-pimaradiene with doxorubicin lowered the IC_50_ of doxorubicin from 4 µM to 1.78 µM. In vivo, both reduced tumor growth and increased necrotic tumor areas. Molecular docking revealed disruption of Bax-Bcl2. **Conclusions:** The findings suggest that *B. grandiflorum* and its major diterpene, epoxy-pimaradiene, exhibit potent anticancer activity, particularly against liver cancer cells. Epoxy-pimaradiene enhances doxorubicin’s efficacy, induces apoptosis, and inhibits tumor progression. Further studies are needed to explore their therapeutic potential.

## 1. Introduction

Throughout human history, medicinal plants have served as a valuable source for discovering and developing new drugs [1]. In modern medicine, natural products have emerged as an important resource for creating new lead compounds and frameworks that aid in drug discovery efforts [2].

Lamiaceae, also known as the mint family, is a large family of flowering plants that includes over 6000 species spread across approximately 236 genera [3]. Native to the Mediterranean region, these plants are known for their herbal or shrub-like growth and distinctively aromatic scent due to their high essential oil content. Along with their volatile oils, Lamiaceae plants are rich in compounds such as tannins, saponins, phenols, flavonoids, and organic acids [4]. They possess valuable biological activities and can potentially be used as antimicrobial, antiviral, antiproliferative, antioxidant, anti-diabetic, anti-hyperlipidemic, anti-inflammatory, antihypertensive, and hepatoprotective agents [5]. The Lamiaceae family, including genera like *Salvia*, *Thymus*, *Origanum*, *Ocimum*, *Melissa*, *Plectranthus*, or *Scutellaria*, exhibits significant anticancer activities, particularly against lung, breast, prostate, and colon cancer cells. Their cytotoxicity, often through apoptosis and effects on angiogenesis, suggests their potential in alternative or complementary cancer therapies [6,7].

*Becium grandiflorum* (Lam). Pic. Serm., a synonym of *Ocimum grandiflorum* [8,9], is a fragrant, perennial woody shrub belonging to the Lamiaceae family and is endemic to Ethiopia. Locally, it is commonly referred to as Tebeb and is traditionally utilized to treat various diseases, including malaria, influenza, spider bites, swellings, and respiratory depression. Two triterpenoidal saponins (beciumecine 1 and 2) were isolated from the root bark of *B. grandiflorum* var. obovatum, along with isothymusin and cirsimaritin from the leaves extract of *B. grandiflorum* [10,11]. Additionally, *B*. *grandiflorum* was reported to exhibit antidiabetic, wound healing, and antimicrobial activity [9,12,13].

Given the numerous cytotoxic diterpenes that have been previously isolated from the Lamiaceae family [14], the aim of this study is to explore the anticancer potential of *B. grandiflorum*, characterize the *n*-hexane fraction using GC-MS, isolate and elucidate its major diterpene, and standardize the *n*-hexane fraction via UPLC-PDA. Additionally, the cytotoxic potential of the *n*-hexane fraction and the major diterpene is evaluated both in vitro and in vivo. This study also investigates their mechanism of action through molecular docking.

## 2. Materials and Methods

### 2.1. Material

The aerial parts of *Becium grandiflorum* (Lam.) pic. Serm. were collected during the flowering stage in the summer of 2020 from Eng. Khalid Al Haddad farm in Giza, Egypt. The identity of the plant material was confirmed by Eng. Therese Labib, botanical specialist and consultant at Orman and Qubba Botanical gardens. A voucher specimen (PHG-P-BG-325) has been preserved in the herbarium of the Pharmacognosy Department, Faculty of Pharmacy, Ain Shams University, Cairo, Egypt. All solvents used were of analytical grade, while those employed in the UPLC and LC-ESI-HRMS assays were of HPLC grade.

### 2.2. Plant Extraction and Fractionation

The aerial parts of *B. grandiflorum* (2 kg) were shade-dried for two weeks. The dried plant materials were macerated in 70% ethanol (10 L) for 48 h at room temperature, followed by filtration. The ethanol extract was then concentrated under reduced pressure at 45 °C yielding a residue of 310 g, which corresponds to a 15.5% yield. A portion of the dried hydroalcoholic extract (277 g) was dissolved in 500 mL distilled water and partitioned with solvents of increasing polarity, providing *n*-hexane (52 g), chloroform (10.5 g), ethyl acetate (8 g), *n*-butanol (25 g), and mother liquor (146 g) fractions which were freeze-dried for subsequent phytochemical and biological analyses.

### 2.3. GC-MS Analysis of N-Hexane Fraction

For the separation and identification of *B. grandiflorum n*-hexane fraction, analytical GC-MS was utilized using a Shimadzu GCMS-QP2010 system equipped with an Rtx-5 MS column (30 m × 0.25 mm i.d. × 0.25 μm film thickness, Restek, Centre County, PA, USA). The procedure was carried out as described by Elhawary et al., (2021) [15]. The chemical constituents were identified by comparing the Kovats index (KI) on the Rtx-5 MS column to the literature and the NIST-11 mass spectral library.

### 2.4. Isolation of the Major Compound from the N-Hexane Fraction

Thirty grams of the *n*-hexane fraction were dissolved in a minimal amount of *n*-hexane and subjected to silica gel column chromatography (Merck, Darmstadt, Germany) (70–230 mesh). The column was eluted with 100% *n*-hexane then *n*-hexane and chloroform with increasing polarity till reaching 100% chloroform. This elution pattern was repeated with ethyl acetate and methanol. A total of 42 fractions were collected and similar fractions were pooled together, resulting in 15 fractions (H-1 to H-15). All fractions were examined by TLC. The chromatograms were sprayed with *p*-anisaldehyde. Fraction H-13 (688 mg) was subjected to silica gel column chromatography (Merck, Darmstadt, Germany) (70–230 mesh). The column was eluted with 100% *n*-hexane, followed by a gradient elution with *n*-hexane and ethyl acetate. Subfraction 10 was eluted with *n*-hexane: ethyl acetate (7:3). The major isolated compound gave a dark purple color after spraying with *p*-anisaldehyde, indicating a diterpene skeleton.

### 2.5. NMR and LC-ESI-HRMS Analysis of the Major Compound from the N-Hexane Fraction

The identity of the compound was determined using ^1^H and ^13^C NMR spectroscopy on a Bruker Avance III system (Fallanden, Switzerland), operating at 400 MHz and 100 MHz, respectively. Samples were dissolved in CDCl_3_ (Sigma Aldrich, Darmstadt, Germany) and analyzed in 3 mm NMR tubes (Bruker). The structure was confirmed using two-dimensional NMR techniques (COSY, HMBC, HSQC). LC-ESI-MS analysis of the major compound was performed on MassHunter workstation B.06.00 (Agilent Technologies, Santa Clara, CA, USA, 2012) using a C_18_ column. The procedure was carried out in accordance with Ibrahim et al., (2022) [16].

### 2.6. Standardization of N-Hexane Fraction Using UPLC-PDA

Standardization of the *n*-hexane fraction was performed using Thermo Fisher UPLC Dionex Ultimate 3000 (Agilent, Santa Clara, CA, USA) equipped with PDA detector and Hypersil Gold ^TM^ C_18_ column (250 × 4.6 mm and 3 μm particle size) as the stationary phase. The mobile phase was isocratic (100% acetonitrile). UV detection was set at 190 nm, with column temperature maintained at 30 °C. A calibration curve of the major compound was established at a concentration range of 31.25–1000 µg/mL; the *n*-hexane fraction concentration was 2 mg/mL [17].

### 2.7. In Vitro Studies

#### 2.7.1. Cell Viability and Cytotoxicity Assays

##### Cell Culture

Human breast cancer (MCF-7 and MDA-MB-231), human hepatocellular cancer (HepG2), and human colon cancer (HCT-116) cell lines were obtained from Nawah Scientific Inc., (Mokatam, Cairo, Egypt). The cells were propagated and maintained under the same process described by Mahmoud et al., (2022) [18].

##### Cell Viability Assays

The cytotoxic potential of *B. grandiflorum* hydroalcoholic extract, *n*-hexane fraction and epoxy-pimaradiene was evaluated using the 3-(4,5-dimethylthiazol-2-yl)-2,5-diphenyltetrazolium bromide (MTT) (Alfa Aesar, Lancashire, UK) assay on MCF-7, MDA-MB-231, HepG2 and HCT-116 cell lines [18]. Briefly, cells were seeded in 96-well plates at a density of 2 × 10^4^ cells per well and allowed to attach overnight at 37 °C and 5% CO_2_. The following day, cells were treated in triplicates with varying concentrations (3.906 to 1000 µg/mL) of the investigated compounds for 72 h. Next, cells were washed three times with sterile 1× PBS before adding 20 µL of 5 mg/mL MTT solution per well followed by incubation at 37 °C for 4 h before the medium was aspirated. The produced formazan crystals were dissolved in 200 µL of isopropanol that had been acidified (0.04 M HCl in 100% isopropanol = 0.073 mL HCL in 50 mL isopropanol) in each well. Using a plate reader (BMG LABTECH^®^ FLUOstar Omega, Ortenberg, Germany), the absorbance was measured at 540 nm with 620 nm serving as the reference wavelength. The 50% inhibitory concentration of cell growth (IC_50_) was calculated using non-linear regression on GraphPad Prism Software (version 6, La Jolla, CA, USA).

Epoxy-pimaradiene was then combined with a known chemotherapeutic agent (doxorubicin). Doxorubicin vials (Adricin^®^, HIKMA pharmaceuticals, London, UK) of 50 mg/25 mL solution for IV injection were purchased from a community pharmacy, Cairo, Egypt. Briefly, cells were seeded in 96-well plates at a density of 3 × 10^5^ cells per well and allowed to attach overnight at 37 °C and 5% CO_2_. The following day, cells were treated in triplicates with varying concentrations of doxorubicin (30–0.23 µM) for 72 h either alone or in combination with epoxy-pimaradiene at 3.24, 4.15, and 5.11 µg/mL, which were calculated as its IC_15_, IC_20_, and IC_25_, respectively. Then MTT assay was performed as previously described.

Combination index (CI) of the major compound and doxorubicin was calculated using the following equation: Combination index = (D)1/(D)1 + (D)2/(Dχ)2 where D1 is IC_x_ of the epoxy-pimaradiene; (Dχ)1 is IC_50_ of epoxy-pimaradiene; D2 is the IC_50_ of epoxy-pimaradiene and doxorubicin in combination while (Dχ)2 represents IC_50_ of doxorubicin alone.

##### Cell Cycle Analysis

HepG2 cells were seeded in 6-well plates at 1 × 10^6^ cells/well and allowed to attach overnight. Next, cells were treated with either 13.40 µg/mL of *n*-hexane fraction, 5.11 µg/mL of epoxy-pimaradiene, 1 µM doxorubicin, or the combination of doxorubicin and epoxy-pimaradiene for 48 h. Cells were then removed from the culture plate, rinsed with cold 1× PBS by centrifugation two times at 280× *g* for 5 min, and then re-suspended in cold PBS and maintained on ice until analysis [19]. A total of 10,000 events were recorded for each sample. The cell cycle distribution was determined using the CytExpert program version 2.4 from Beckman Coulter, Brea, CA, USA.

##### Annexin V/Propidium Iodide (PI) Apoptosis Assay

In T-25 flasks, 1 × 10^6^ HepG2 cells were seeded and allowed to adhere overnight. The following day, cells were exposed to 13.40 µg/mL of *n*-hexane fraction, 5.11 µg/mL of the major compound, 1 µM doxorubicin or the combination of doxorubicin and the major compound for 72 h. Following the use of trypsin to separate the cells, the samples were rinsed with cold 1× PBS, centrifuged twice at 280× *g* for 5 min, then re-suspended in cold PBS, and maintained on ice until analysis [19]. For each sample, a minimum of 10,000 events were collected. CytExpert software (Beckman Coulter, Brea, CA, USA) was used for data analysis.

##### Determination of Levels of Apoptotic Proteins Using ELISA

HepG2 cells, 5 × 10^6^, were maintained in T-75 flasks for 24 h. Then the media were substituted with DMEM medium as untreated control or supplemented with 13.40 µg/mL of *n*-hexane fraction, 5.11 µg/mL of 18-epoxy-pimara 8(14),15 diene (major compound), 1 µM doxorubicin or the combination of doxorubicin and 18-epoxy-pimara 8(14),15 diene and incubated for 72 h. Protein extraction and sample total protein concentrations were determined using BCA assay [19].

Next, protein levels of BAX, BCL-2, and caspases 3,8, and 9 were determined using human BAX ELISA kit (Catalog no. E4511-100), BioVision, Milpitas, CA, USA; human BCL-2-like Protein 1 ELISA kit (Catalog no. E4035hu), BT Lab, Shanghai, China; human Caspase-3 ELISA kit (Catalog no. K4221-100), BioVision, Milpitas, CA, USA; human Caspase-7 ELISA kit (Catalog no. E2257Hu), BT Lab, Shanghai, China; human Caspase-8 ELISA kit (Catalog no. E-EL-H0659), Elabscience, Houston, TX, USA and human Caspase-9 ELISA kit (Catalog no. NBP1-83734), Novus Biologicals, Centennial, CO, USA, respectively, as described by the manufacturers’ protocols.

### 2.8. In Vivo Experimentation

All in vivo experiments were conducted after the approval of the ethical review committee of The Faculty of Pharmacy, The British University in Egypt, Cairo, Egypt (approval number EX-2316) in accordance with the Animals (Scientific Procedures) Act 1986/ASPA Amendment Regulations 2012.

#### 2.8.1. Experimental Design

The experimental design was in conformance with ARRIVE guidelines. A total of forty-two female Swiss Albino mice (22–30 g, 6–7 weeks old) were used in this study. Mice were inbred at the animal facility of The British University in Egypt. They were housed under standard laboratory conditions of temperature (25 ± 2 °C) and relative humidity (55–60%) with free access to water and food. An Ehrlich ascites carcinoma (EAC) cells solid phase tumor model was used as previously described [18]. Briefly, two mice were implanted with EAC cells intraperitoneally (I.P) to induce ascites. After 7 days, the ascetic fluid was aspirated and diluted 1:10 in saline and injected intramuscularly (I.M) into the left flank of forty mice to induce a solid tumor mass. After 10 days and once tumors were detectable in mice thighs, they were randomly subdivided into 5 groups (n = 8 per group). Randomization was performed manually by randomly allocating the cages to the respective treatment groups. Mice in which no palpable tumors were detected were excluded from the study (n = 2). Group 1 (untreated control) received saline; group 2 (DOX) received 4 mg/Kg doxorubicin (as positive control) IP once per week; group 3 (HEX 50 mg/Kg) received 50 mg/kg of *B. grandiflorum n*-hexane fraction IP 5 times a week; group 4 (HEX 100 mg/Kg) received 100 mg/kg of *B. grandiflorum n*-hexane fraction IP 5 times a week and group 5 (Epoxy-pimaradiene) received 2 mg/kg IP 5 times a week. Gebremeskel et al. (2020) evaluated the acute oral toxicity of *Becium grandiflorum* using OECD Guideline 425 and found that it was well tolerated up to 5000 mg/kg with no toxicity [9]. Given this safety margin and the unknown oral bioavailability, 50 mg/kg and 100 mg/kg were chosen as pharmacologically relevant doses to balance efficacy and safety in our in vivo model.

Mice were weighed regularly, and any adverse events or mortality incidents were reported [20]. Tumor dimensions (length and width) were measured twice per week using a digital caliper and tumor volume was calculated using the following equation:

Tumor volume (mm^3^) = 0.52 × (minor axis)^2^ × (major axis).

After 21 days, mice were sacrificed by cervical dislocation and tumors were collected and split into 2 halves: one half was stored at −20 °C until further processing and the second half was added to a tube containing 10% formalin-saline until histological examination.

#### 2.8.2. Histopathological Examination

The fixed tumors were dehydrated and placed in paraffin wax before being cut into thin 4 μm sections and de-waxed, rehydrated, and finally stained with hematoxylin and eosin. Tumor tissues were examined using light microscopy. The area of necrotic tissue in the tumors was determined by analyzing non-overlapping microscopic fields per tumor mass per sample using a high-resolution camera and Leica application module (Leica Microsystems GmbH, Wetzlar, Germany). To measure the mean percentage of necrotic tissue relative to the entire tumor mass, a specialized automated histomorphometry unit operated by a consultant histologist was used to analyze all tissue samples from each group with high precision [21].

#### 2.8.3. Statistical Analysis

For the analysis of in vitro and in vivo samples, blinding was employed. Samples IDs were replaced by codes which were not shared with the investigator undergoing analyses. The data obtained from the study were represented as mean ± standard deviation (SD) or standard error of mean (SEM). They were analyzed using one or two-way analysis of variance (ANOVA) followed by Tukey-Kramer test for post hoc analysis. The significance of the statistical results in this research was determined by a *p*-value of less than 0.05. Statistical analyses and graphical representations were performed using GraphPad Prism version 6.00 for Windows (La Jolla, CA, USA).

### 2.9. In Silico Disruption of Bax-Bcl2 Interaction by Terpenoid-Induced Bcl2 Conformational Changes

All molecular modeling experiments were conducted using Schrödinger Suite (Schrödinger). Protein files were obtained from the Protein Data Bank (PDB) [22]. The co-crystal structure for venetoclax with Bcl-2 (PDB-ID: 600 K) [23] and that of Bax BH3 peptide with Bcl-2 (PDB-ID: 2XA0) were used for computational experiments. Protein files were imported into the Protein Preparation Wizard and prepared using default settings (filling of sidechains, optimization of hydrogen-bonding assignments and protein minimization) [24]. Ligand files for venetoclax and the major isolated compound were generated as SDF files using ChemDraw version 16.0 (CambridgeSoft) and input into Schrödinger Suite for preparation. Ligands were prepared using LigPrep module utilizing the OPLS_2005 force field with retention of input chiralities.

#### 2.9.1. Induced-Fit Docking

Induced-fit docking was carried out using the Induced Fit Docking module of Schrödinger Suite [25]. The binding site was defined by selection of venetoclax as a reference for the binding site. Docking settings were set to dock ligands of <15 Å in length. Residues within 5 Å of the docked poses were rendered flexible for Prime refinement of docked poses. Glide redocking was carried out following refinement using extra precision settings for most accurate results.

#### 2.9.2. Data Analysis and Graphics

Docking scores from extra precision docking and induced-fit docking are generated as XP-scores in free energy units (kcal/mol). Conversion of docking scores into computed dissociation constant (K_D_) was conducted using the following equation:

$$∆G=RT\ln K_{D}$$

Docked poses were visualized using Maestro version 9.0 and PyMol version 2.6 (Schrödinger). All graphical figures were generated using PyMol.

## 3. Results

### 3.1. GC-MS Analysis of N-Hexane Fraction of B. grandiflorum

Characterization of the *n*-hexane fraction using GC-MS analysis (Figure S1) resulted in the tentative identification of 21 metabolites representing 87.67% of the total fraction composition. The identified chemical constituents belonged to various chemical classes including hydrocarbons, fatty acid ethyl esters, oxygenated diterpenes, lignans, and phytosterols (Table 1). Oxygenated diterpenes (pimarene-type) were the major class, accounting for 70.49% of the total composition of the *n*-hexane fraction. The chief identified compounds were 18-epoxy-pimara 8(14),15 diene (the major isolated compound) (27.90%), Pimara-8(14),15-dien-3-ol (14.02%), 3- *α*-Hydroxy-manool (10.92%), retinol (6.39%), and Pimara-8(14),15-dien-3-one (6.14%) (Figure S2). The high abundance of oxygenated diterpenes in *B. grandiflorum n*-hexane fraction was in line with Roozbehani *et al.,* (2021) concluding that diterpenoids were the chief chemical class in the *O. basilicum* L. *n*-hexane fraction obtained from Iran [26]. In contrast, Mustafa et al., (2019) found that alcohols (39.09%) and fatty acids (38.87%) constitute the major classes of compounds in the *O. americanum n*-hexane fraction cultivated in Sudan [27].

### 3.2. Isolation and Identification of the Major Compound from N-Hexane Fraction

**Figure 1:**
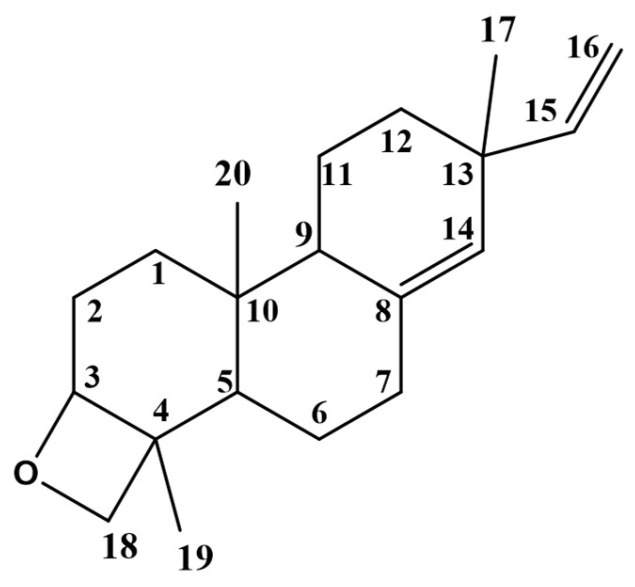
Chemical structure of 18-epoxy-pimara-8(14),15-diene (Epoxy-pimaradiene).

The major compound was isolated as colorless needle crystals (Figure 1). The HRESIMS analysis showed a molecular ion at *m/z* 287.23803 [M+H]^+^ (calculated for 287.23694) equivalent to the molecular formula of oxygenated diterpene (C_20_H_30_O). The ^1^H-NMR and ^13^C-NMR spectral data at (400 and 100 MHz, CDCl_3_) are summarized in Table 2. The ^1^H-NMR spectrum (Figure S3) pattern showed proton signals similar to *ent-*pimara-8(14),15- diene that were previously reported by Bromann (2014) [28]. A singlet olefinic proton signal was detected at δ_H_ 5.25, corresponding to H-14. A doublet of doublet proton signal integrated for one proton was detected at δ_H_ 5.78, corresponding to H-15 showing vicinal coupling with H-16_a_ and H-16_b_. Two doublet of doublet signals appeared at δ_H_ 4.96 ppm (*J* = 1.5 and 17.5 Hz) and 4.87 ppm (*J* = 1.5 and 10.6 Hz) corresponding to the olefinic protons H-16_a_ and H-16_b_. Three singlet proton signals were assigned for three methyl groups at δ_H_ 1.05, 0.94, and 0.89 ppm, corresponding to H-17, H-19, and H-20, respectively. The absence of a fourth methyl group at H-18 in distinction from *ent-*pimara-8(14), 15-diene suggests that this methyl group was involved in oxetane ring formation, which was confirmed by the downfield shift of H-3 and H-18 protons. The ^13^C-NMR spectrum (Figure S4) confirmed the ^1^H-NMR spectrum with the presence of only three methyl signals assigned to C-17, C-19, C-20 at δ_C_ (25.97, 11.54, 15.50 ppm), respectively. In addition, a downfield methylene signal detected at δ_C_ 72.01 ppm was assigned to C-18, which was not detected in the previously reported *ent-*pimara-8(14),15-diene [28]. Moreover, a noticeable downfield shift of the tertiary C-3 at δ_C_ 77.07 ppm (instead of the methylene at δ_C_ 39.10) suggested that the oxetane ring is linked to C-3. Furthermore, a downfield shift of C-2 from δ _C_ 19.00 to 27.25 ppm was detected. The suggested structure was confirmed by 2D-NMR. The COSY spectrum (Figure S5) displayed correlation between the neighbor vinyl protons H-15 and H-16 and another correlation was established between the proton H-3 and the proton H-18, which confirm that C-3 and C-18 are involved in the oxetane ring. The assignment of C-3, C-14, C-15, C-16, and C-18 was made by observation of their respective correlation on the HSQC (Figure S6) experiment. Two cross peaks correlate the methylene protons at δ_H_ 3.44 and 3.70 ppm to the downfield carbon C-18. Another cross peak correlates the methine proton at δ_H_ 3.70 ppm to the downfield carbon at C-3 confirming its substitution. The absence of CH_3_ at C-18 confirms that it was involved in the oxetane ring as a methylene group. The structure was further resolved by the HMBC (Figure S7) experiment, where the site of attachment was solved by the cross peak of methylene protons at C-18 to the downfield carbon resonance at C-3. Furthermore, the HMBC correlation was closely related to the peak signals confirming the proposed structure e.g., the correlation between the olefinic carbon C-14 (δ_C_ 129) and both olefinic H-15 (δ_H_ 5.78) and the methyl H-17 (δ_H_ 1.05). The presence of the oxetane group in diterpenes (Diepoxy-*ent*-kauranes) of the Lamiaceae family was previously documented [29]. Thus, the isolated compound is identified as 18-epoxy-pimara-8(14),15-diene (Epoxy-pimaradiene). This compound was used as a standard reference for the standardization of the *n*-hexane fraction.

### 3.3. UPLC-PDA Standardization of N-Hexane Fraction of B. grandiflorum

Quantitative estimation of 18-epoxy-pimara-8(14),15-diene (epoxy-pimaradiene) in the *n*-hexane fraction was undertaken using UPLC-PDA. The chromatogram of the *n*-hexane fraction was recorded at 210 nm (Figure S8) and showed a major peak at R_t_ = 3.29 min corresponding to epoxy-pimaradiene. Figure S9 represents the chromatogram of epoxy-pimaradiene. For the assessment of the linear range, a serial dilution of the major compound was prepared at concentrations of 31.25, 62.5, 125, 250, 500, 1000 µg/mL. The calibration curve of epoxy-pimaradiene (Figure S10) confirmed the linearity of the method within the working range. Regression equations and the regression coefficient (R^2^) were calculated as follows (y = 0.8034x + 4.5827) and (R^2^= 0.9998). It was observed that each 1 mg of the *n*-hexane fraction contains 0.3027 mg of 18-epoxy-pimara-8(14),15-diene. The validation parameters for the HPLC method, including linearity, LOD, LOQ, recovery, and precision are presented in Table 3.

### 3.4. In Vitro Screening of *B. grandiflorum*

#### 3.4.1. Cell Viability and Cytotoxicity Assessment

The cytotoxic effect of *B. grandiflorum* hydroalcoholic extract, the *n*-hexane fraction, and the major isolated compound, 18-epoxy-pimara-8(14),15-diene (Epoxy-pimaradiene) were investigated against various cell lines: human breast cancer cells (MCF-7 and MDA-MB-231), human colon cancer cells (HCT-116), and human hepatocellular carcinoma (HepG2). The results (Table 4 and Figure S11) revealed varying degrees of cytotoxic activity across the tested samples, with a notable impact on HepG2 cells. Subsequent biological studies focused on this cell line. Notably, the *n*-hexane fraction and epoxy-pimaradiene exhibited the highest cytotoxic activity. Additionally, MCF-7 breast cancer cells appeared more sensitive to treatment compared to the more invasive MDA-MB-231 cells.

**Figure 2:**
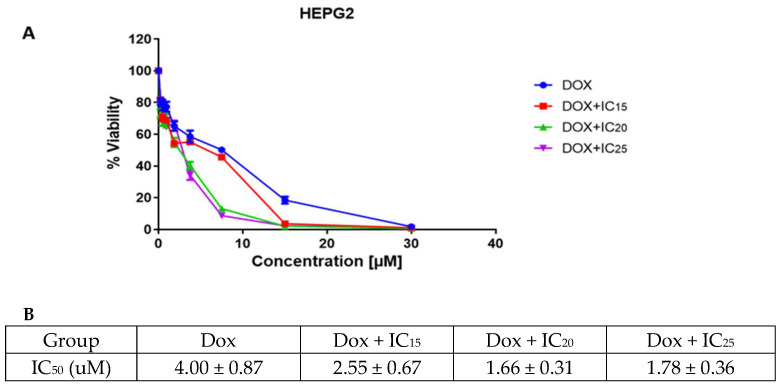
Results of the cell viability and cytotoxicity assessment. (**A**) cell viability after 72 h of incubating HepG2 cells with doxorubicin as a monotherapy or in combination with IC_15_, IC_20_ and IC_25_ of epoxy-pimaradiene. (**B**) table showing the calculated IC_50_ of doxorubicin monotherapy and in combination with IC_15_, IC_20_ and IC_25_ of epoxy pimaradiene. Data was represented as mean ± SD.

Next, the in vitro cytotoxic effect of doxorubicin and epoxy-pimaradiene combination was evaluated on HepG2. Cells were treated with different concentrations of doxorubicin and incubated for 72 h to estimate its IC_50_. Then epoxy-pimaradiene was added to doxorubicin at different fixed concentrations (IC_15_, IC_20_, and IC_25_). Doxorubicin caused a dosedependent decrease in the cell viability of HepG2 cells with a calculated IC_50_ of 4.00 ± 0.87 µM. Interestingly, epoxy pimaradiene potentiated the anti-cancer effect of doxorubicin. The IC_50_ was reduced to 2.55 ± 0.67, 1.66 ± 0.31, and 1.78 ± 0.36 µM when doxorubicin was combined with the IC_15_, IC_20,_ and IC_25_, respectively, of epoxy pimaradiene with calculated combination indices of 0.9, 0.76, and 0.873, respectively. These findings suggest a synergistic interaction between the standard anticancer drug and epoxy pimaradiene (as shown in Figure 2).

#### 3.4.2. Results of Cell Cycle Analysis

**Figure 3:**
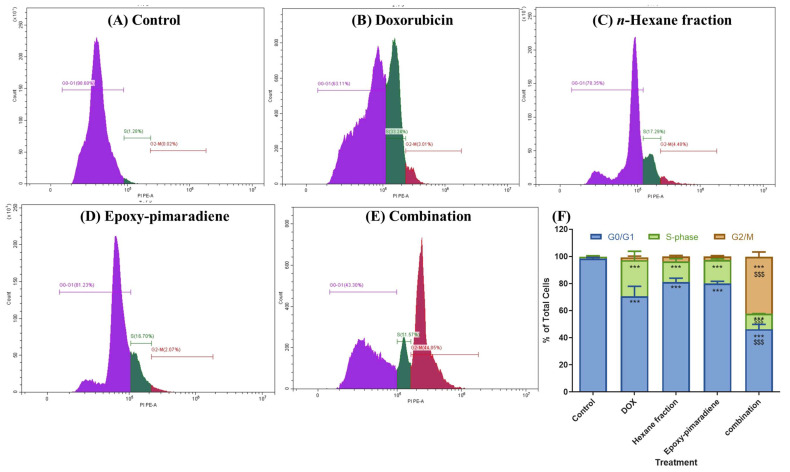
Results of the Cell cycle analysis showing the effect of doxorubicin, *n*-hexane fraction, epoxy-pimaradiene as monotherapies and a combination of doxorubicin + epoxy-pimaradiene on the cell cycle progression of HepG2 cells. (**A**–**E**) Histograms represent the distribution of cell population among each cell cycle stage. (**F**) Bar chart demonstrating the percentage of total cells detected in each phase (G0/G1, S-phase and G2/M). *** *p* ≤ 0.0005 relative to untreated control and $$$ *p* ≤ 0.0005 relative to the comparison between the combination and doxorubicin, one-way ANOVA, Tukey’s post-Hoc. Values are expressed as mean ± SD.

Cell cycle analysis was performed to investigate the effect of doxorubicin, *n*-hexane fraction, epoxy-pimaradiene, and a combination of doxorubicin + epoxy-pimaradiene on the progression of the cell cycle. Results (Figure 3) revealed significant differences (*p* ≤ 0.0005) between the untreated control and the four tested treatment groups with (98.52% ± 0.77) of the cells at the G_0_/G1 phase and (1.36% ± 0.71) of the cells at the S-phase in the untreated control. Doxorubicin caused a shift in the cell population to the S-phase (26.66% ± 6.5) similar to the *n*-hexane and epoxy-pimaradiene, which also caused a shift to the S-phase but to a lesser extent (15.14% ± 2.11 and 17.30 % ± 0.85), respectively. Interestingly the combination therapy led to a G2/M arrest (42.04% ± 3.54). Upon comparing the combination to doxorubicin alone, a significant difference was observed at the three cell phases (G_0_/G1, S and G2/M) (*p* ≤ 0.0005). These findings demonstrate the ability of the combination to alter the cell cycle which in turn can affect the cancer progression.

#### 3.4.3. Results of Annexin V/PI Apoptosis Assay

**Figure 4:**
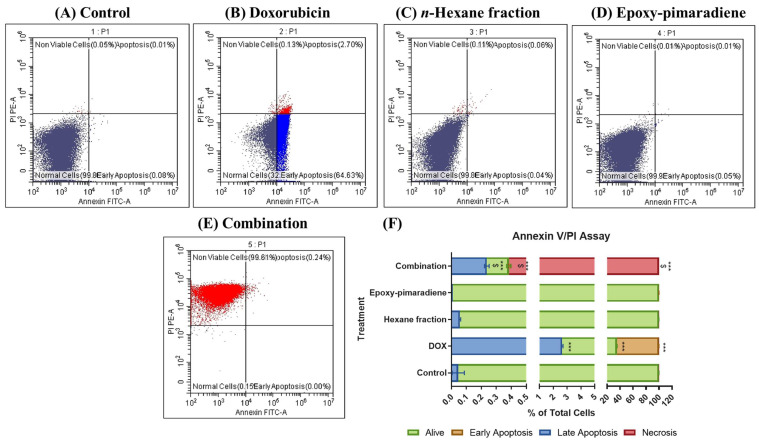
Results of Annexin V/PI apoptosis assay showing the effect of doxorubicin, *n*-hexane fraction, epoxy-pimaradiene as monotherapies and a combination of doxorubicin + epoxy-pimaradiene on HepG2 cells. (**A–E**) Dot plots representing Annexin V-FITC *against* PI signal. (**F**) Stacked bar chart showing the percentage of cells detected in each quadrant. n = 3 wells/treatment *** *p* ≤ 0.0005 versus the untreated control and $ *p* ≤ 0.0005 versus doxorubicin, one-way ANOVA, Tukey’s post hoc. Values are expressed as mean ± SD.

The Annexin V/PI apoptosis assay was applied to investigate the role of apoptosis in the cytotoxic effect of the four tested groups. Results showed that in the untreated control, *n*-hexane fraction, and epoxy-pimaradiene most cell populations were alive with 99.84% ± 0.07, 99.8% ± 0.04, and 99.9% ± 0.02, respectively, with no significant difference between those groups (Figure 4). Meanwhile, in doxorubicin, most of the cells were in an early apoptosis state (64.52% ± 0.12). The combination of doxorubicin + epoxy-pimaradiene showed significant differences versus the control and doxorubicin (*p* ≤ 0.0005). In the combination, the percentage of cells in necrosis was increased significantly to 99.61% ± 0.005 (*p* ≤ 0.0005) with a significant decrease in the % of live cells (0.14% ± 0.01).

#### 3.4.4. The Effect of *N*-Hexane Fraction, Epoxy-Pimaradiene, Doxorubicin, and the Combination Therapy on the Key Apoptotic Protein

**Figure 5:**
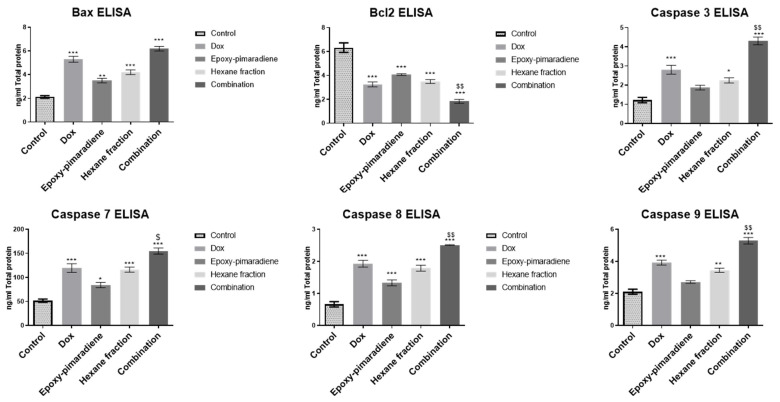
ELISA results of BAX, BCL-2, caspase 3, 7, 8, and 9 on HepG2 following treatment with doxorubicin, epoxy-pimaradiene, *n*-hexane fraction, and the combination of doxorubicin and epoxy-pimaradiene. All the tested groups significantly induced BAX and inhibited BCL-2 in comparison to the control. The combination of doxorubicin with epoxy-pimaradiene showed a significant difference in the reduction in BCL-2 in comparison with doxorubicin monotherapy. Doxorubicin, hexane fraction, and the combination showed significant increases in all caspases (3, 7, 8, and 9) while epoxy-pimaradiene showed a significant increase in caspase 7 and caspase 8 and no significant difference in caspase 3 and 9 in comparison to the control. Upon comparing the combination therapy to doxorubicin monotherapy, it showed significant differences in BCL-2, caspase 3, 7, 8, and 9. (n = 3 * *p* ≤ 0.05, ** *p* ≤ 0.005, *** *p* ≤ 0.0005 versus the untreated control and $ *p* ≤ 0.05 and $$ *p* ≤ 0.005 versus doxorubicin, ordinary One-way ANOVA, Tukey’s post hoc. Values are expressed as mean ± SEM).

The effect of the four tested treatment groups was evaluated on the key apoptotic protein markers (BAX, BCL-2, and caspase 3,7, 8, and) 9 which are vital for stopping tumor progression and the activation of the intrinsic and/or extrinsic apoptotic pathways [30]. The results demonstrated that all the tested groups significantly induced the pro-apoptotic marker BAX and inhibited the anti-apoptotic marker BCL-2 in comparison to the control (*p* ≤ 0.0005). Moreover, the combination of doxorubicin with epoxy-pimaradiene showed significant difference in the reduction in BCL-2 in comparison with doxorubicin monotherapy (*p* ≤ 0.005). Furthermore, doxorubicin, *n*-hexane fraction and the combination showed a significant increase in all caspases (3, 7, 8, and 9) (*p* ≤ 0.005 and 0.0005 versus the control) while the epoxy-pimaradiene showed a significant increase in caspase 7 (*p* ≤ 0.05) and caspase 8 (*p* ≤ 0.0005) only and no significant difference in caspase 3 and 9 in comparison to the control. It was also revealed that upon comparing the combination therapy to doxorubicin monotherapy, it showed a significant decrease in BCL-2 and an increase in caspase 3,7,8, and 9 (*p* ≤ 0.05 and 0.005) (Figure 5). In conclusion, *n*-hexane fraction and epoxy-pimaradiene showed powerful cytotoxic activity against all tested cell lines, specifically, HepG2 cells, and shifted the cell cycle towards the S-phase compared to the control with a significant induction of the key apoptotic markers. Additionally, the combination of epoxy-pimaradiene with doxorubicin potentiated its anticancer activity with a powerful induction of apoptosis.

### 3.5. In Vivo Anticancer Efficacy of B. grandiflorum on Ehrlich Ascites Carcinoma

#### 3.5.1. Effect of the Tested Samples on the Tumor Volume

**Figure 6:**
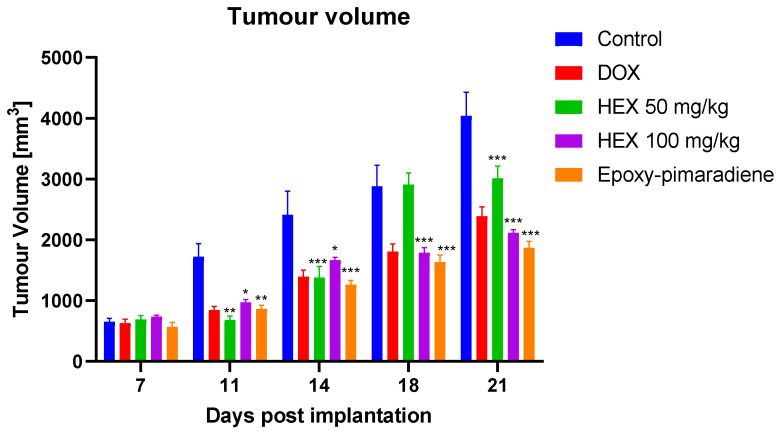
The effect of doxorubicin, *n*-hexane fraction and epoxy-pimaradiene on the tumor volume of EAC bearing mice. (n = 8 per group * *p* ≤ 0.05, ** *p* ≤ 0.005, *** *p* ≤ 0.0005 versus the untreated control, two-way ANOVA, Tukey’s Post-Hoc. Values are expressed as mean ± SEM).

The presented bar chart illustrated in Figure 6 shows the tumor volume measured on days 7, 11, 14, 18, and 21 post EAC cells implantation. It was observed that on day 7, tumor volumes started increasing in all treatment groups, indicating the success of the model but with no significant differences amongst the different groups. The untreated control group showed a consistent and significant increase in tumor volume throughout the duration of the experiment. Nonetheless, all treatment groups demonstrated a significant reduction in tumor progression compared to the untreated control group (*p* ≤ 0.005). The greatest tumor regression was observed with treatment group 5 (epoxy-pimaradiene) (*p* ≤ 0.0005). This was followed by group 4 (*n*-hexane fraction 100 mg/kg). No significant difference in the tumor volume was observed between the reference doxorubicin and *n*-hexane fraction and epoxy-pimaradiene. These findings support the potential anti-neoplastic activity of the *B. grandiflorum* hexane fraction and the major isolated compound.

#### 3.5.2. Body Weight of Tumor-Bearing Mice

**Figure 7:**
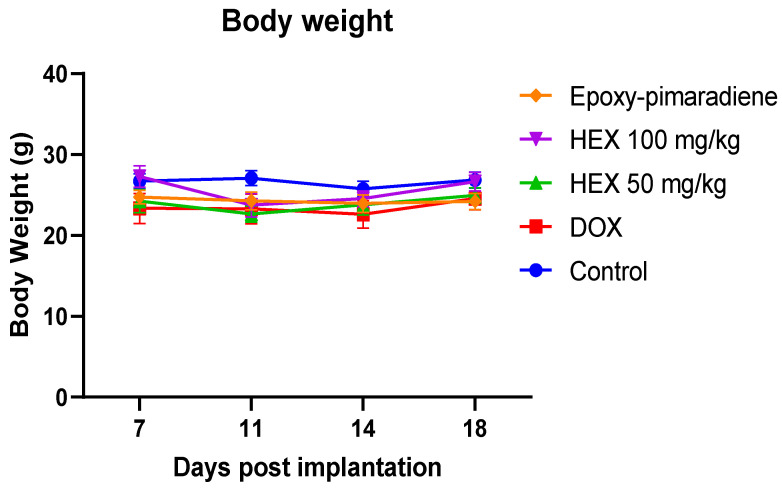
The effect of doxorubicin, *n*-hexane fraction, and epoxy-pimaradiene on the body weight of tumor-bearing mice. No significant difference was observed between all treatment groups. (n = 8, two-way ANOVA, Tukey’s post-hoc. Values are expressed as mean ± SEM).

The body weight of the EAC tumor-bearing mice (Figure 7) showed no significant difference detected between all treatment groups throughout the experiment in comparison to the untreated control group. This finding supports the relative safety of the *B. grandiflorum n*-hexane fraction and epoxy-pimaradiene to be used as a potential anti-neoplastic complementary therapy.

#### 3.5.3. Tumor Histopathological Examination

**Figure 8:**
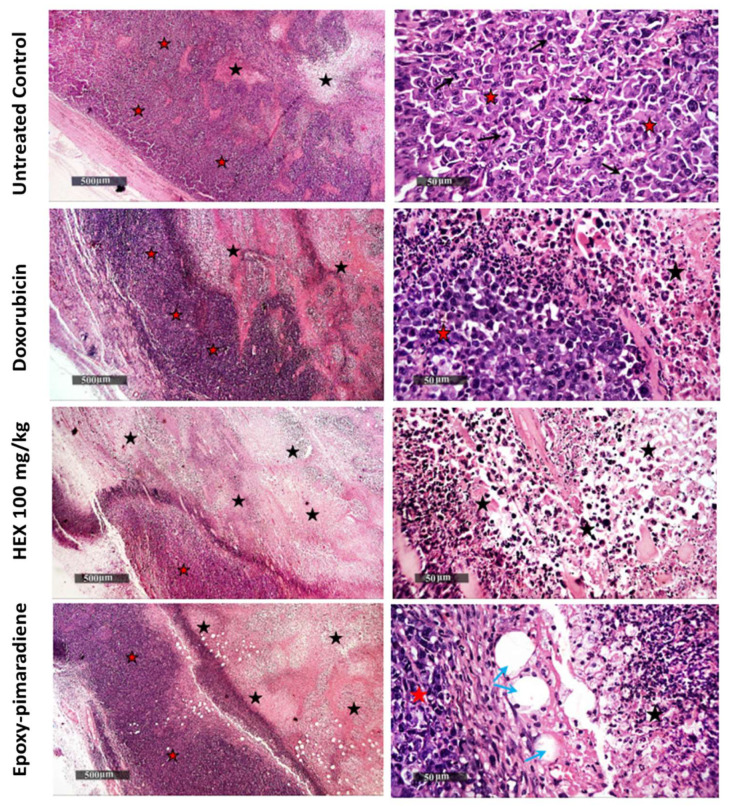
Photomicrographs of hematoxylin and eosin-stained tumor tissues (H&E stain), magnification power 50× and 500×. Black arrows: areas of basophilic, pleomorphic, and live tumor cells with conspicuous nucleoli throughout the outside, middle, and interior zones of the tumor mass, abundant mitotic. Black stars: islets of necrotic tissue debris with infiltrates of mononuclear cells beneath the outer fibrous capsule. Red stars: living tumor cell sheets with minimal mitotic figures. Blue arrows: intra-tumor mass vacuolization.

As shown in Figure 8, untreated control tumor samples showed massive areas of basophilic, pleomorphic, and live tumor cells with conspicuous nucleoli throughout the outside, middle, and interior zones of the tumor mass, abundant mitotic figures with some islets of necrotic tissue debris with distinct records of infiltrates of mononuclear cells beneath the outer fibrous capsule. While doxorubicin samples demonstrated a moderate rise in the number of apoptotic bodies and middle necrotic tissue areas mass, nonetheless, living tumor cell sheets were displayed with minimal mitotic figures. Additionally, there were numerous recordings of inflammatory cell infiltrates with little neovascularization. Since the tumor volume of mice treated with the hexane fraction given at 50 mg/kg showed similar results to the 100 mg/Kg group, it was excluded from the histological analysis. Hexane 100 mg/kg samples exhibited the highest degree of anti-tumor activity, with an extensive core necrotic mass and thinner exterior sheets of viable cells with the lowest number of mitotic figures. Epoxy-pimaradiene samples revealed moderately elevated records of apoptotic bodies and a necrotic central mass area with multiple intra-tumor mass vacuolization. Persistent moderate findings of outer viable thick sheets of tumor cells were also demonstrated with the least mitotic figures. These findings support the potent anti-tumor activity of the hexane fraction and the isolated compound.

**Figure 9:**
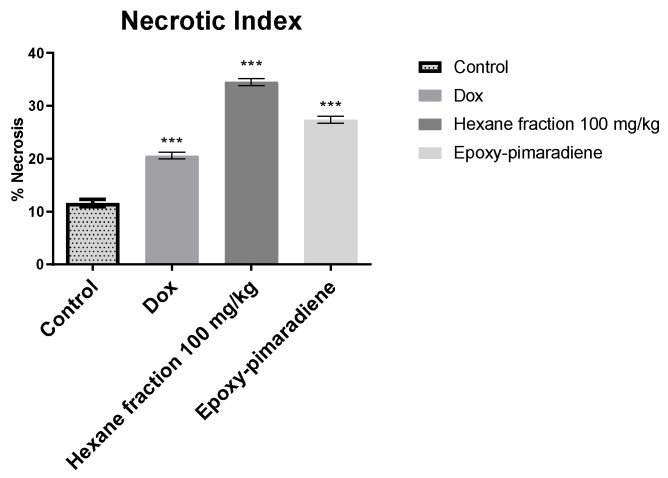
Percent of tumor necrosis for histopathological samples (untreated control, doxorubicin, *n*-hexane fraction 100 mg/kg, and epoxy-pimaradiene. *** significant at *p* < 0.0001 compared to untreated control using one-way ANOVA followed by Tukey Kramer post hoc test. Values are expressed as mean ± SEM).

Considering the inverse relationship between tumor necrosis and tumor progression, the percentage of necrosis was evaluated for the processed tumor samples. As presented in Figure 9, the *n*-hexane fraction at a concentration of 100 mg/kg showed the highest necrotic index with 34.51% of necrosis, followed by epoxy-pimaradiene (27.4%) then doxorubicin (20.61%) and untreated control samples (11.65%). All the tested groups showed a significant difference *p* < 0.0001 as compared to the untreated control. All these findings confirm the in vivo and in vitro antitumor activity of the hexane fraction and the iso lated diterpene.

### 3.6. In Silico Disruption of Bax-Bcl2 Interaction by Terpenoid-Induced Bcl-2 Conformational Changes

**Figure 10:**
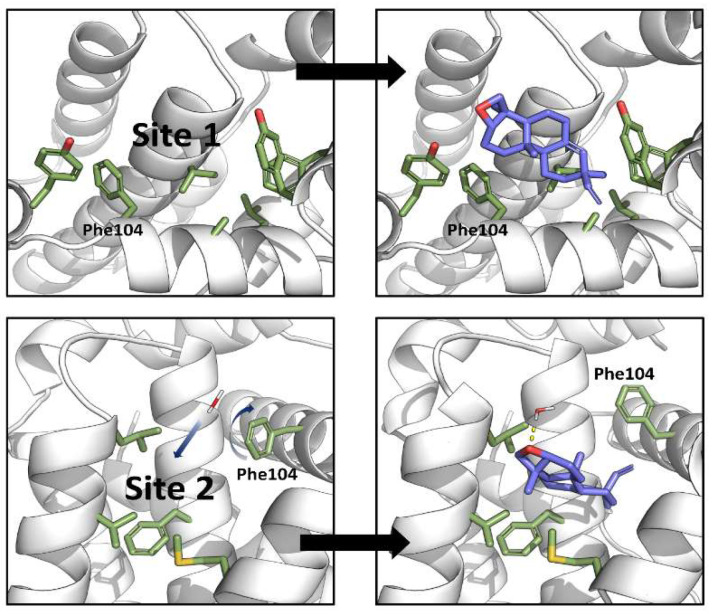
Computational studies revealed two binding sites for epoxy-pimaradiene within the BH3-peptide-binding site of Bcl-2 by extra-precision docking (**top panel**) and induced-fit docking (**bottom panel**). Blue arrows highlight structural changes that take place upon binding of epoxy-pimaradiene to site 2 (motion of water and rotation of the side chain of Phe104).

**Figure 11:**
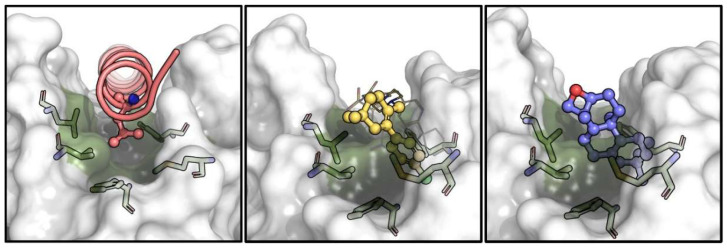
Comparison of the binding pose for Leu63 of Bax BH3 peptide (PDB-ID: 2XA0, pink, (**left panel**)), *p*-chlorophenylcyclohexenyl of venetoclax (PDB-ID: 600 K, yellow, (**middle panel**)) and epoxy-pimaradiene (docked, purple, (**right panel**)) within site 2 of Bcl-2 (green). Hydrophobic residues lining this site are shown as sticks and the general surface of Bcl-2 is displayed in semi-transparent white.

Biochemical and cell-based assays of epoxy-pimaradiene show it induces apoptosis by inhibiting Bcl-2, a protein that prevents apoptosis by interacting with the pro-apoptotic protien Bax [31]. To understand this mechanism, computational methods were used to study the binding of epoxy-pimaradiene to the BH3-binding site of Bcl-2 utilizing the co-crystal structure of venetoclax (PDB-ID: 600 K) [32]. Initial molecular docking (extra precision) identified a shallow, hydrophobic pocket originally occupied by Leu70 of the Bax BH3 peptide (site 1) but found it unsuitable due to the compound’s hydrophobic nature (Figure 10, top panel). This pocket is also surrounded by several hydrophilic contacts which stabilize the interaction of venetoclax in its crystal structure [23]. Induced-fit docking revealed a deeper, more hydrophobic pocket (site 2) surrounded by residues Phe104, Met115, Val133, Leu137, and Phe153 (Figure 10, bottom panel). In the venetoclax-Bcl-2 co-crystal structure, this pocket is occupied by the highly hydrophobic *p*-chlorophenylcyclohexenyl moiety (Figure 11). This pocket aligns well with the compound’s size and hydrophobicity, yielding a superior XP-score (−6.9 kcal/mol) compared to site 1 (−3.3 kcal/mol), indicating a 500-fold higher affinity.

**Figure 12:**
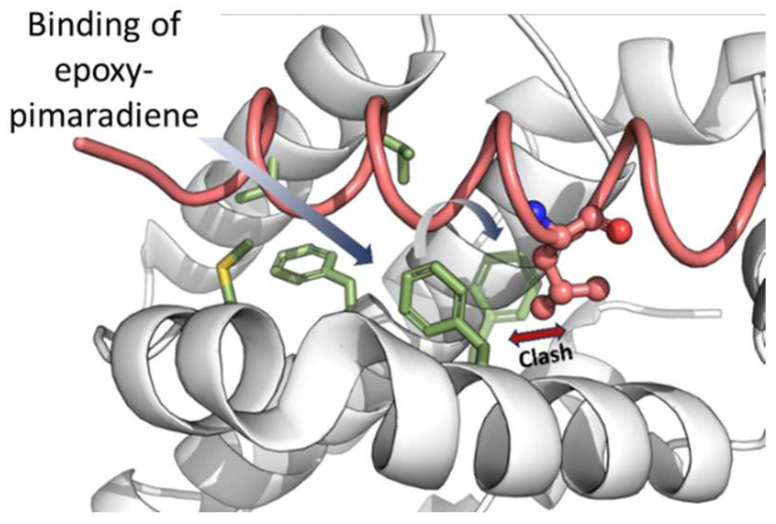
The effect of binding of epoxy-pimaradiene on the conformation of Phe104. Upon binding, the sidechain of Phe104 rotates (semi-transparent green sticks) to occupy site 1 and occludes Leu70 of Bax BH3 peptide (pink) from binding to site 1. This motion would also prevent a second molecule of epoxy-pimaradiene from binding to site 1 while site 2 is simultaneously occupied.

Binding to site 2 (that of Bax-Leu63) causes the compound to be fully buried within the hydrophobic residues, with its oxetane ring exposed to the solvent. A crystallographic water molecule (ID: Wat447) forms a hydrogen bond with the compound. Crucially, the binding induces a conformational change in Phe104, allowing the compound to fit into site 2 and blocking site 1 (Figure 12). This conformational change is essential for the compound’s binding and function, suggesting that epoxy-pimaradiene primarily binds to site 2, preventing Bax from binding to site 1, thereby exerting its apoptotic effect.

In conclusion, our computational models reveal two binding sites for epoxy-pimaradiene where binding takes place primarily in site 2 with computationally determined micromolar dissociation constants. Binding to site 2 occludes site 1 towards the BH3 peptide and towards the binding of a second molecule of epoxy-pimaradiene simultaneously. This may explain the significant apoptotic and cytotoxic potential of epoxy-pimaradiene.

## 4. Discussion

Cancer, a genetic and multifaceted disease characterized by uncontrolled cell growth, significantly impacts global health with about 11 million new cases annually, affecting various tissues and organs, and often resulting from genetic changes induced by carcinogens [33,34].

The GC-MS analysis of the *n*-hexane fraction of *B*. *grandiflorum* identified 21 compounds, predominantly oxygenated diterpenes. Among these were pimara-8(14), 15-dien-3-ol, pimara-8(14),15-dien-3-one, and 18-epoxy-pimara-8(14),15-diene, the latter being the major isolated compound. Other notable compounds included 3-*α*-hydroxy-manool and retinol. Pimarane diterpenes isolated from fungal and plant sources showed promising biological activities including cytotoxic and anticancer [14,35,36]. Kongwaen et al., (2023) evaluated the cytotoxic potential of fifteen isolated isopimarane diterpenoids including two new isopimaranes namely, trihydroxy isopimaradiene and dihydroxy isopimaradiene against ten human cancer cell lines including HepG2 and MDA-MB-231. The tested isopimaranes showed a mild cytotoxic potential against cancer cell lines with a very low cytotoxicity against the normal cell line [37]. Moreover, *ent*-8(14),15-pimaradien-3*β*-ol and *ent*-pimara-8(14),15-dien-19-oic acid isolated from *Aldama arenaria* showed moderate cytotoxic activity against childhood leukemia cell lines HL-60 [38].

In addition, retinol (Vitamin A) was found to exhibit cytotoxic activity through the induction of morphological and proliferative alterations by the activation of multiple kinases [39]. Moreover, retinol and all trans retinoids showed anti-tumor activity against breast cancer through affecting certain growth-factor pathways [40]. The presence of oxygenated diterpenes, specifically pimarane diterpenes and retinol, as major constituents of the hexane fraction likely explains the potent cytotoxic activity observed in the *n*-hexane fraction.

In addition, combining doxorubicin with 18-epoxy-pimara-8(14),15-diene significantly reduced doxorubicin’s IC_50_ in HepG2 cells, indicating a synergistic effect that could allow for lower, less toxic doses of doxorubicin. Combining natural compounds like terpenes with doxorubicin can enhance chemotherapy efficacy through multi-target effects, improved pharmacokinetics, resistance interference, and toxicity reduction [41,42,43].

This study evaluated the anticancer potential of the *B. grandiflorum n*-hexane fraction and 18-epoxy-pimara-8(14),15-diene on the cell cycle progression and induction of apoptosis in HepG2 cells, human hepatocellular carcinoma. Cell cycle regulation, crucial for controlled cell growth, is often disrupted in cancer [44,45,46,47]. In the present study, we evaluated the effect of doxorubicin, *n*-hexane fraction, epoxy-pimaradiene, and a combination of doxorubicin and epoxy-pimaradiene on the cell cycle of HepG2 cells. The combination treatment significantly increased the proportion of cells in the G2/M phase (42.04% ± 3.54) compared to the untreated control (0.03% ± 0.02) and doxorubicin alone (2.12% ± 0.89). To further validate these findings, future studies should investigate the expression of key G2/M transition regulators such as cyclin B1, CDK1, and p21, to gain deeper insights into the molecular mechanisms underlying the observed G2/M arrest [48,49].Previous studies on terpenes like carnosol and ursolic acid showed their capability to arrest the cell cycle. Carnosol can induce cell cycle arrest through affecting AMP-activated protein kinase [50]. Ursolic acid induces its anticancer activity by arresting the cell cycle at the G1 phase in WA4 mammary cells [51,52]. Moreover, oridonin, another diterpene, induced G2/M phase arrest and increased apoptosis in HepG2 cells [53]. Combined treatments of doxorubicin and oridonin in human breast carcinoma cells (MDA-MB-231 and MCF-7) showed synergistic effects, enhancing nuclear condensation and apoptosis [54]. Moreover, extracts from *Ocimum sanctum* and *Ocimum gratissimum* also demonstrated cell cycle arrest at the G0/G1 phase in head and neck squamous cell carcinoma (HNSCC) [55]. Furthermore, *O. gratissimum* aqueous extract induced cell cycle arrest in hepatocellular carcinoma SK-Hep1 and HA22T cells at the sub-G1 phase [56]. These findings suggest that diterpenes in *Ocimum* extracts may activate cell cycle regulatory pathways, inhibiting cancer cell proliferation. This aligns with the observed effects in this study, supporting the potential of B. grandiflorum and its compounds as anticancer agents.

The role of apoptosis in the cytotoxic activity of *B. grandiflorum* against the HepG2 cell line was further investigated using an Annexin V/PI apoptosis assay and ELISA to assess apoptotic protein markers (BAX, BCL-2, and caspases 3, 7, 8, and 9). Four treatment groups (doxorubicin, hexane fraction, epoxy-pimaradiene, and the combination) were examined. The combination treatment resulted in 99.61% necrosis, significantly higher than the control (0.07%) and doxorubicin alone (0.11%). All tested groups significantly induced BAX and inhibited BCL-2 compared to the control, with the combination treatment showing a greater reduction in BCL-2 than doxorubicin alone. The Annexin V/PI assay showed increased necrosis in the combination treatment group; however, our ELISA data (Figure 5) indicate significant BAX upregulation, BCL-2 downregulation, and caspases activation, supporting apoptosis as the primary mechanism. The observed necrosis is likely secondary necrosis [57] rather than direct cytotoxicity. To further distinguish between apoptosis-induced necrosis and primary necrosis, future studies using LDH release assays [58] or caspase inhibition (e.g., Z-VAD-FMK) [59] are recommended. Additionally, cytotoxicity assays on non-cancerous cell lines could help confirm selectivity and rule out non-specific toxicity. Previous studies on *Ocimum* extracts and diterpenes show they can induce apoptosis in cancer cells by affecting BCL-2 family proteins and caspases. For instance, *O. sanctum* ethanolic extract activated caspases 3 and 9 and downregulated BCL-2 in LNCaP prostate cancer cells, leading to DNA fragmentation and cell death [60]. *O. gratissimum* extract induced apoptosis in A549 lung cancer cells by activating caspases and modulating BAX and BCL-2 levels. [61]. Additionally, diterpenes like clerodane and cembranoids have been shown to induce apoptosis and cell cycle arrest in various cancer cell lines through caspase activation [62,63]. The apoptotic activity of *B. grandiflorum* is likely due to its high diterpene content, which may enhance the cytotoxic effects of doxorubicin. Combining doxorubicin with diterpenes could be a promising cancer treatment approach, potentially enhancing efficacy and reducing side effects. Further research is needed to fully understand the mechanisms and therapeutic potential of this combination.

To further confirm the in vitro assay results, the in vivo antitumor potential of the *B. grandiflorum* hexane fraction (50 and 100 mg/kg) and epoxy-pimaradiene (2 mg/kg) was tested against doxorubicin using the Ehrlich ascites carcinoma (EAC) model, widely used in cancer research. The study showed significant tumor volume reduction in the treatment groups compared to the untreated control group, confirming the antitumor activity of both the hexane fraction and epoxy-pimaradiene. Additionally, there was no significant change in the body weight of the mice, indicating no adverse effects on their metabolic rate, food intake, or overall health [64]. Toxicity assessment was based on body weight and behavioral observations, with no adverse effects noted. These findings are consistent with Gebremeskel et al. (2020), who reported no oral toxicity for *Becium grandiflorum* at doses up to 5000 mg/kg following OECD Guideline 425 [9]. Nonetheless, future studies should further evaluate potential systemic toxicities (e.g., renal, hepatic, CNS, or cardiovascular effects) or the lack thereof. Histopathological examinations revealed that the hexane fraction and the isolated diterpene exhibited strong antitumor activity, with epoxy-pimaradiene showing intra-tumor mass vacuolization, a morphological change associated with cell death or necrosis [65]. These findings align with previous studies highlighting the cytotoxic and antitumor activity of diterpenes, which have shown the ability to inhibit tumor growth and exhibit cytotoxic effects against EAC cells [14,66].

## 5. Conclusions

Phytochemical investigation of the *n*-hexane fraction of *Becium grandiflorum* led to the isolation of a new diterpene, 18-epoxy-pimara-8(14),15-diene, with GC-MS analysis revealing that oxygenated diterpenes are the major compounds. Both the *n*-hexane fraction and 18-epoxy-pimara-8(14),15-diene, either alone or in combination with doxorubicin, exhibited significant anticancer potential. This study is the first to explore the anticancer properties of *B. grandiflorum*, suggesting it could be a valuable treatment for cancer. Further research is needed to study the pharmacokinetics and impact on the lifespan of 18-epoxy-pimara-8(14),15-diene to better understand its biological effects and optimal dosing for clinical use.

## Figures and Tables

**Table 1:** Chemical profile of *n*-hexane fraction prepared from *B. grandiflorum* aerial part using GC-MS.

Peak #	Component	Molecular Formula	Molecular Weight	RT (Min)	Relative Percentile (%)	KI	
						Obsd.	Lit.
1	Hexahydrofarnesyl acetone	C_18_H_36_O	268	31.686	0.23	1841	1842
2	1-Eicosene	C_20_H_40_	280	33.288	0.36	1923	1986
3	Ethyl palmitate	C_18_H_36_O_2_	284	34.74	0.86	1995	1995
4	Ethyl linolenate	C_20_H_34_O_2_	306	38.122	0.61	2173	2171
5	Unidentified	-	-	38.475	0.42	2192	-
6	3- *α*-Hydroxy-manool	C_20_H_34_O_2_	306	39.35	**10.92**	2241	2273
7	4-Epidehydroabietol	C_20_H_30_O	286	39.625	0.34	2257	2326
8	Pimara-8(14),15-dien-3-ol	C_20_H_32_O	288	39.945	**14.02**	2275	2253
9	*ent*-Kaurenol	C_20_H_32_O	288	40.240	**3.83**	2291	2302
10	Isopimarol	C_20_H_32_O	288	40.805	**3.51**	2324	2305
11	Verticillol	C_20_H_34_O	290	41.050	0.82	2338	2106
12	Unidentified	-	-	41.145	0.18	2344	-
13	Retinol	C_20_H_30_O	286	42.370	**5.36**	2417	2453
14	Kauren-19-oic acid	C_20_H_30_O_2_	302	42.815	0.53	2444	2358
15	Unidentified	-	-	43.030	1.51	2457	-
16	Retinol	C_20_H_30_O	286	43.195	1.03	2467	2453
17	Unidentified	-	-	43.590	0.47	2491	-
18	Communic acid	C_20_H_30_O_2_	302	43.785	0.75	2503	2404
19	Unidentified	-	-	44.205	0.7	2530	-
20	Sandaracopimaric acid	C_20_H_30_O_2_	302	44.455	1.73	2546	2282
21	18-epoxy-pimara- 8(14),15-diene	C_20_H_30_O	286	44.665	**27.90**	2559	-
22	Pimara-8(14),15-dien-3-one	C_20_H_30_O	286	45.265	**6.14**	2597	2279
23	Unidentified	-	-	45.710	0.57	2626	-
24	Unidentified	-	-	46.995	0.29	2712	-
25	Unidentified	-	-	47.155	0.57	2722	-
26	Unidentified	-	-	47.365	0.45	2737	-
27	Sesamin	C_20_H_18_O_6_	354	53.605	**3.94**	3193	3150
28	Unidentified	-	-	54.520	2.52	3258	-
29	Unidentified	-	-	54.965	0.43	3288	-
30	2-methyloctacosane	C_29_H_60_	408	55.130	1.59	3300	2866
31	Unidentified	-	-	55.380	0.7	3315	-
32	δ7,25-Stigmastadienol	C_29_H_48_O	412	55.755	2.8	3338	3325
33	Unidentified	-	-	55.900	1.69	3346	-
34	*Gamma.*-Sitosterol	C_29_H_50_O	414	56.045	0.4	3355	3351
35	Unidentified	-	-	56.395	1.18	3376	-
36	Unidentified	-	-	56.555	0.65	3386	-
Total identified %		87.67					
Number of identified components		21					
Hydrocarbons %		1.95					
Sesquiterpenes %		0.23					
Oxygenated diterpenes %		70.49					
Fatty acids %		1.47					
Sterols %		3.2					
Lignans %		3.94					
Others %		6.39					

KI _obsd._: Kovats indices evaluated experimentally on Rtx-5 MS column relative to C8–C28 *n*-alkanes series. KI _lit_: Reported Kovats indices. Bolded numbers are the highest relative percentiles % among all compounds.

**Table 2:** ^1^H-NMR and ^13^C-NMR, COSY and HMBC of 18-epoxy-pimara-8(14),15-diene.

C/H Position	δ_H_, Multi.	δ_C_, Type	COSY	HMBC
1	1.72, m (1H) 1.14, m (1H)	36.9, CH_2_		
2	1.62–1.72 m (2H)	27.25 CH_2_		
3	3.7 m (1H)	77.07 CH		
4	-	42.1 C		C-18
5	1.14, m (1H)	48.6 CH		
6	1.40–1.45, m (2H)	22.4 CH_2_		
7 *a*	2.26, m (1H)	35.7 CH_2_		
7 *β*	2.05, m (1H)			
8	-	136.9 C		
9	1.75, m (1H)	50.5 CH		
10	-	37.7 C		
11	1.57–1.52 m (2H)	18.7 CH_2_		
12	1.50–1.44 m (2H)	34.4 CH_2_		
13	-	37.4 C		
14	5.25, s (1H)	129.04 CH		C-15-C-17
15	5.78 dd (1 H) *J*_1_ 10.6 Hz and *J*_2_ 17.5 Hz	148.8 CH	H-16	C-16
16 a	4.96, dd (1H) *J*_1_= 1.5 Hz and *J*_2_ = 17.5 Hz	110.15 CH_2_	H-15	
16 b	4.87, dd (1H) *J*_1_ = 1.5 Hz and *J_2_* = 10.6 Hz		H-15	
17	1.05, s (3H)	25.97 CH_3_		
18	3.44, d (1H) 3.7, d (1H)	72.01 CH_2_	H-3	C-3, C-19
19	0.94, s (3H)	11.54 CH_3_		C-18
20	0.89, s (3H)	15.50 CH_3_		

**Table 3:** Validation parameter of UPLC-PDA method.

Validation Parameter	Result
Linearity (R^2^)	0.9998
Limit of detection (LOD) (µg ml^−1^)	10.46
Limit of quantification (LOQ) (µg ml^−1^)	31.70
Recovery % (mean± SD)	100.58 ± 1.64
Precision (RSD%) Intra-day Inter-day	0.07 0.08

**Table 4:** IC_50_ of *B. grandiflorum* hydroalcoholic extract, *n*-hexane fraction and epoxy-pimaradiene on human breast cancer, human colon cancer, and human liver cancer cell lines.

Cell Line	IC_50_ (µg/mL)		
	Hydroalcoholic Extract	*N*-Hexane Fraction	Epoxy-Pimaradiene
MCF-7	43.54 ± 9.19	20.29 ± 2.16	23.06 ± 2.02
MDA-MB-231	-	89.10 ± 10.05	123.20 ± 16.21
HCT-116	40.51 ± 5.25	15.54 ± 1.64	10.61 ± 0.61
HepG2	19.85 ± 2.34	13.75 ± 1.05	11.45 ±1.20

## Data Availability

The data that support the findings of this study are openly available from the corresponding authors upon reasonable request.

## Supplementary Materials

The following supporting information can be downloaded at: https://www.mdpi.com/article/10.3390/nu17071164/s1, Figure S1: GC-MS chromatogram of *n*-hexane fraction; Figure S2: Major identified metabolites in *n*-hexane fraction using GC-MS; Figure S3: ^1^H-NMR spectrum 18-epoxy-pimara- 8(14),15-diene in CDCl_3_ (400 MHz); Figure S4: ^13^C–NMR spectrum of 18-epoxy-pimara-8(14),15-diene in CDCl_3_ (100 MHz); Figure S5: ^1^H-^1^H-COSY spectrum of 18-epoxy-pimara- 8(14),15-diene in CDCl_3_; Figure S6: HSQC spectrum of 18-epoxy-pimara- 8(14),15-diene in CDCl_3_; Figure S7: HMBC spectrum of 18-epoxy-pimara- 8(14),15-diene in CDCl_3_; Figure S8: UPLC-PDA chromatogram of *B. grandiflorum n*-hexane fraction; Figure S9: UPLC-PDA chromatogram of 18-epoxy-pimara- 8(14),15-diene; Figure S10: Calibration curve of 18-epoxy-pimara- 8(14),15-diene; Figure S11: Cytotoxicity of hydroalcoholic extract, *n*-hexane fraction, and compound 2 on (A) MCF-7, (B) MDA-MB-231, (C) HCT-116, and (D) HepG2 cell lines.

## Abbreviations

The following abbreviations are used in this manuscript:

HCT-116	Human colon cancer cell line
MCF-7	Human breast cancer cell line
MDA-MB-231	Human triple-negative breast cancer cell line
HepG2	Human liver cancer cell line
DMEM	Dulbecco’s Modified Eagle Medium
BCA	Bicinchoninic Acid
BCL-2	B-Cell Lymphoma 2
