# Supplementary material for: Phytochemical Analysis and In Vivo Anticancer Effect of Becium grandiflorum: Isolation and Characterization of a Promising Cytotoxic Diterpene
Source: Nutrients. 2025 Mar 27;17(7):1164. doi: 10.3390/nu17071164 (PMC11990180; doi:10.3390/nu17071164)
Supplement: Supplementary file 1 [file nutrients-17-01164-s001.zip › nutrients-3502657-supplementary.pdf]

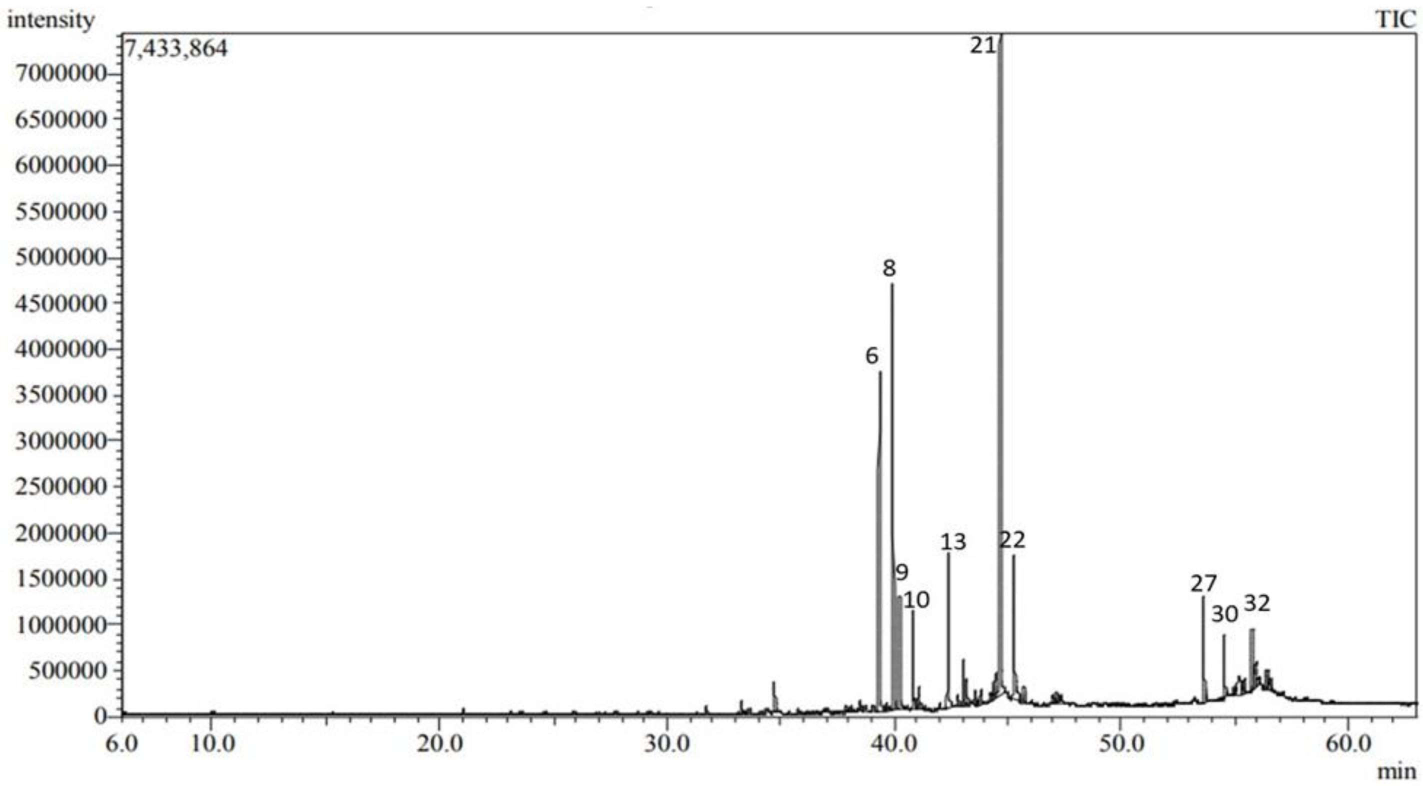

Figure S1. GC-MS chromatogram of *n*-hexane fraction.

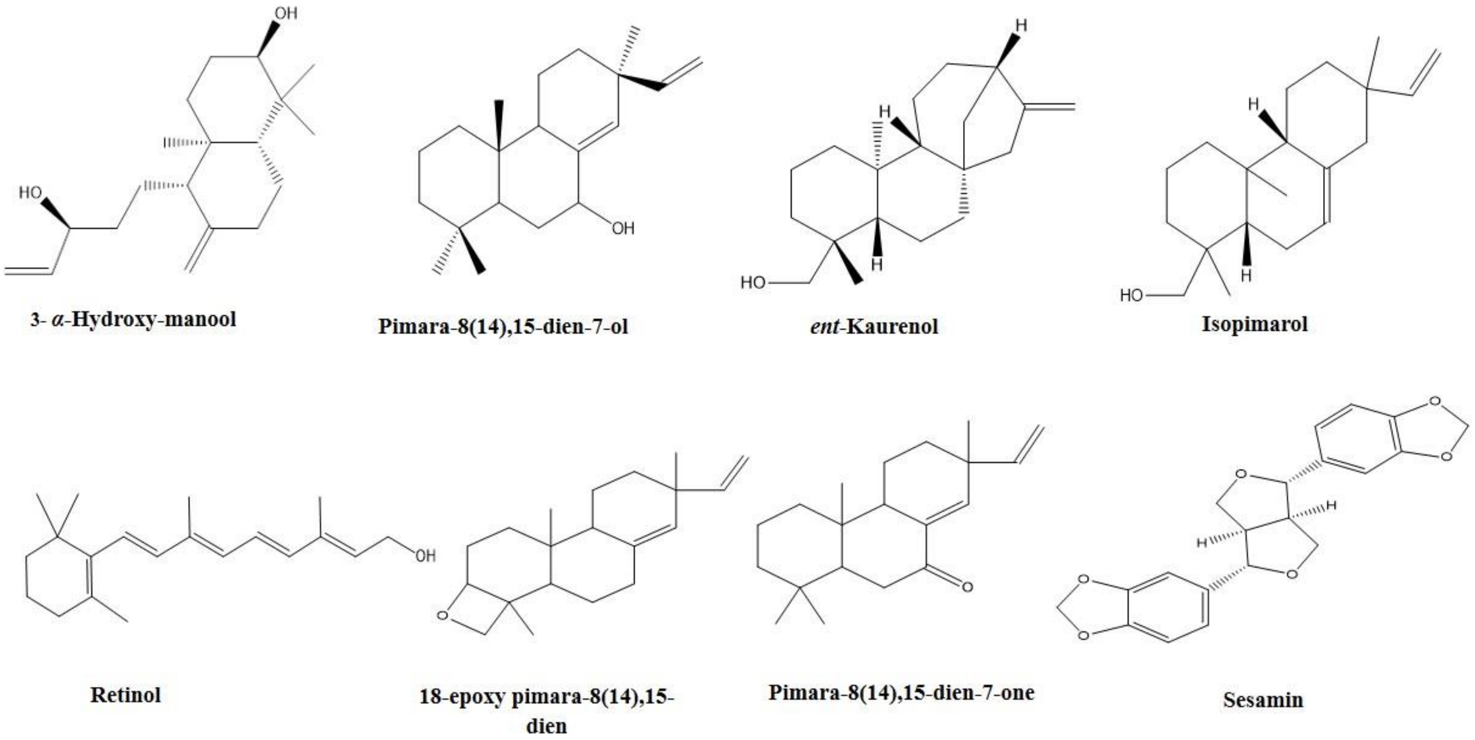

Figure S2. Major identified metabolites in *n*-hexane fraction using GC-MS.

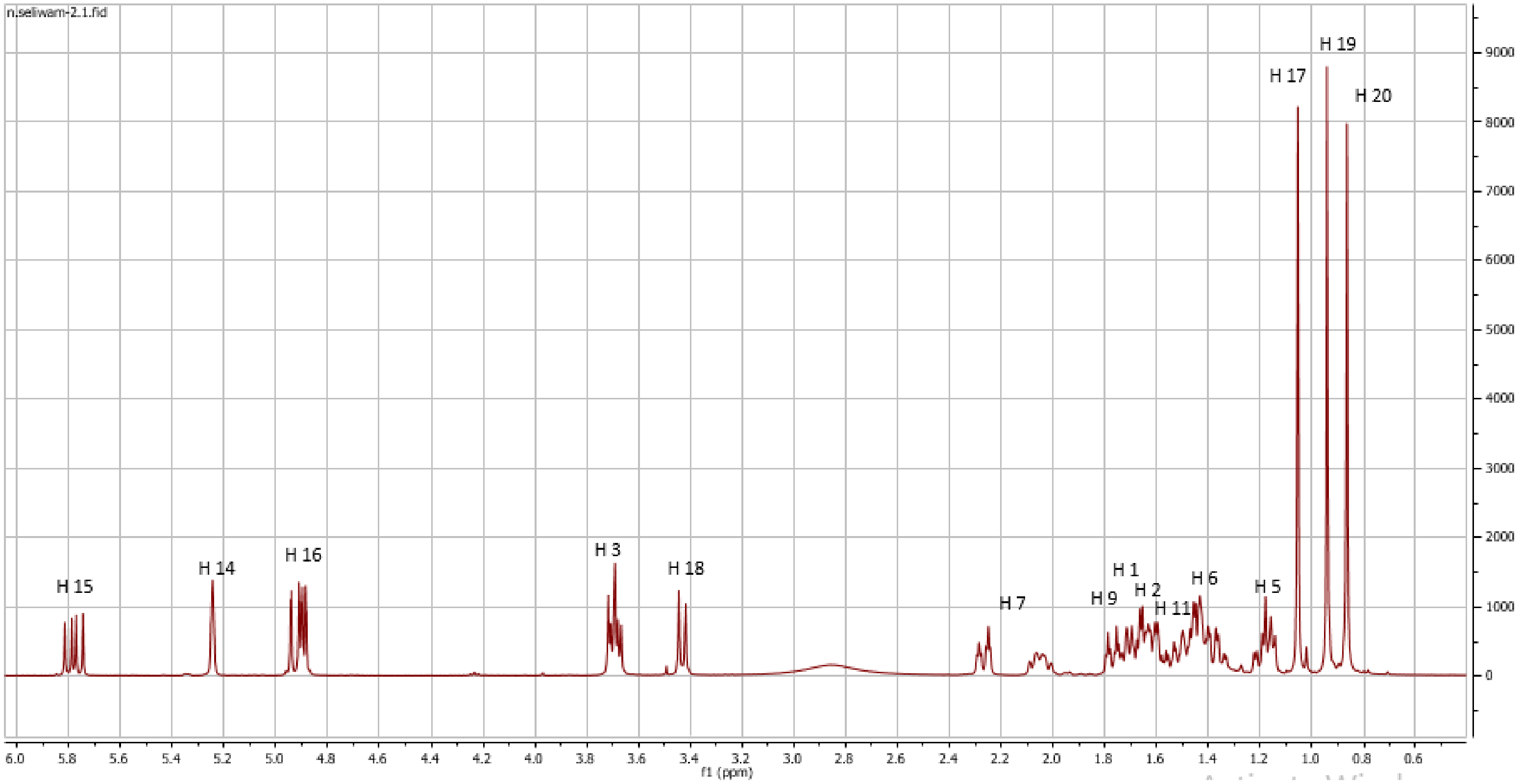

Figure S3. ^{1}H-NMR spectrum 18-epoxy-pimara- 8(14),15-diene in $CDCl_3$ (400 MHz).

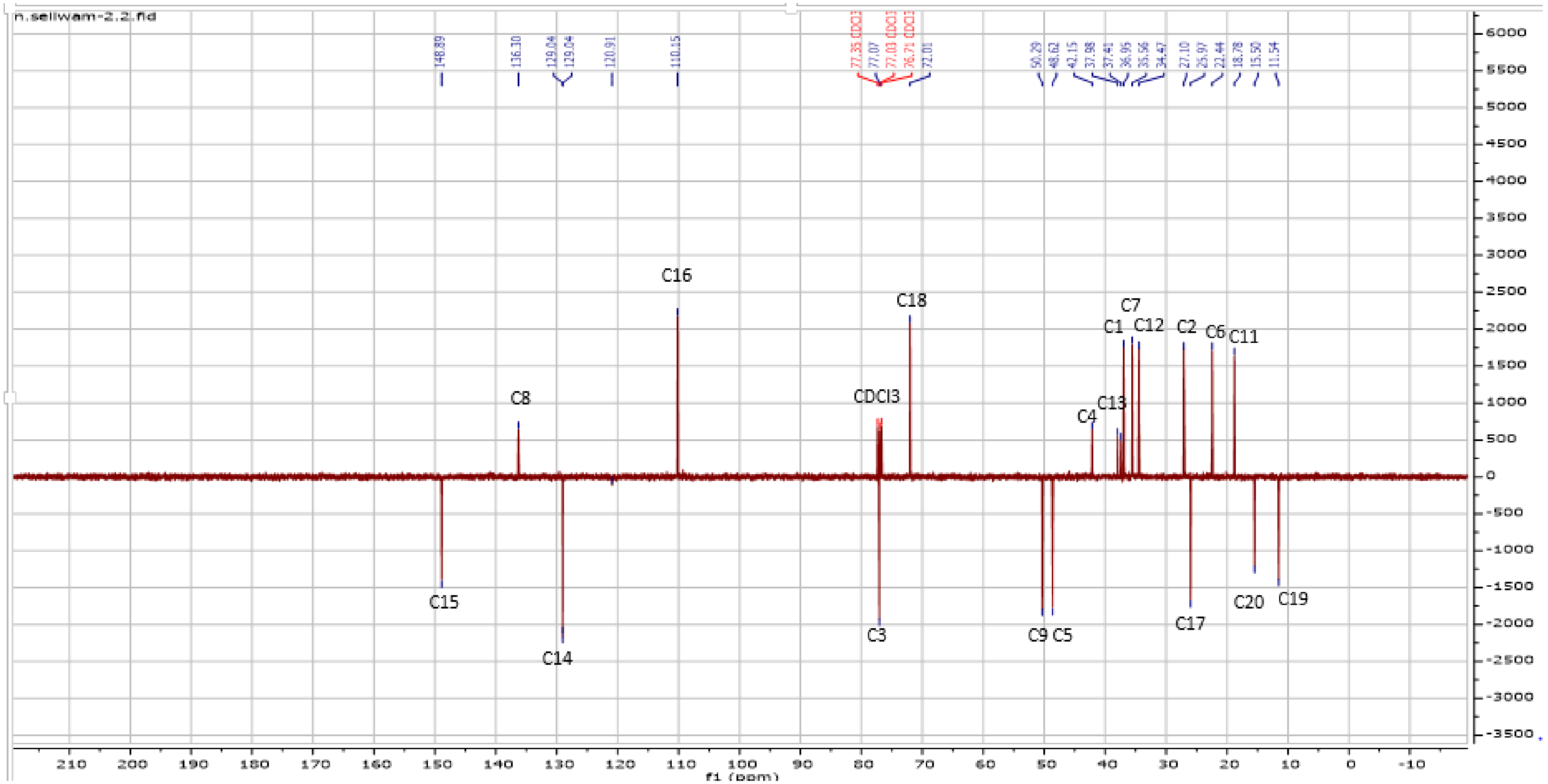

Figure S4. ^{13}C –NMR spectrum of 18-epoxy-pimara- 8(14),15-diene in $CDCl_3$ (100 MHz).

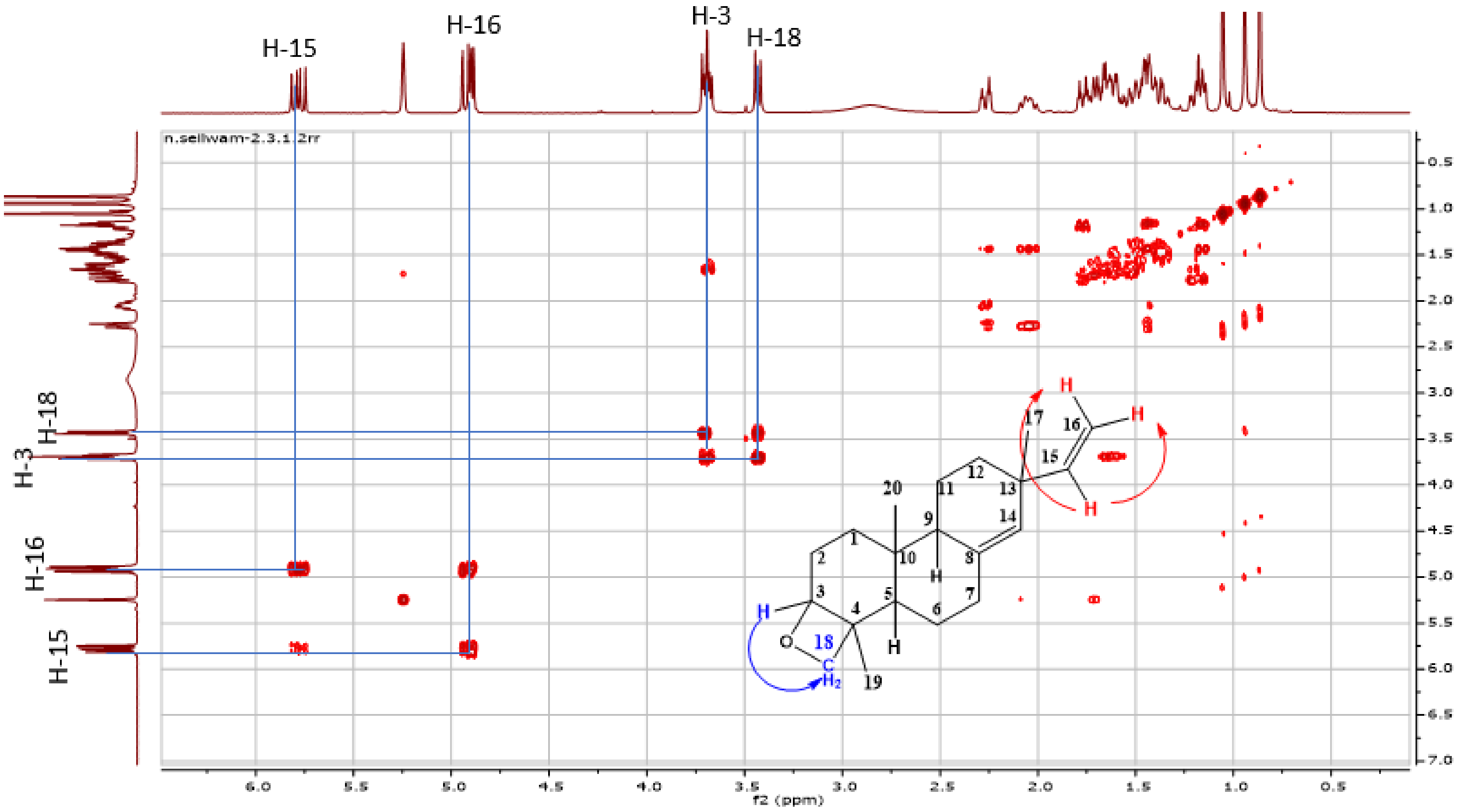

Figure S5. ^1H-^1H-COSY spectrum of 18-epoxy-pimara- 8(14),15-diene in $CDCl_3$.

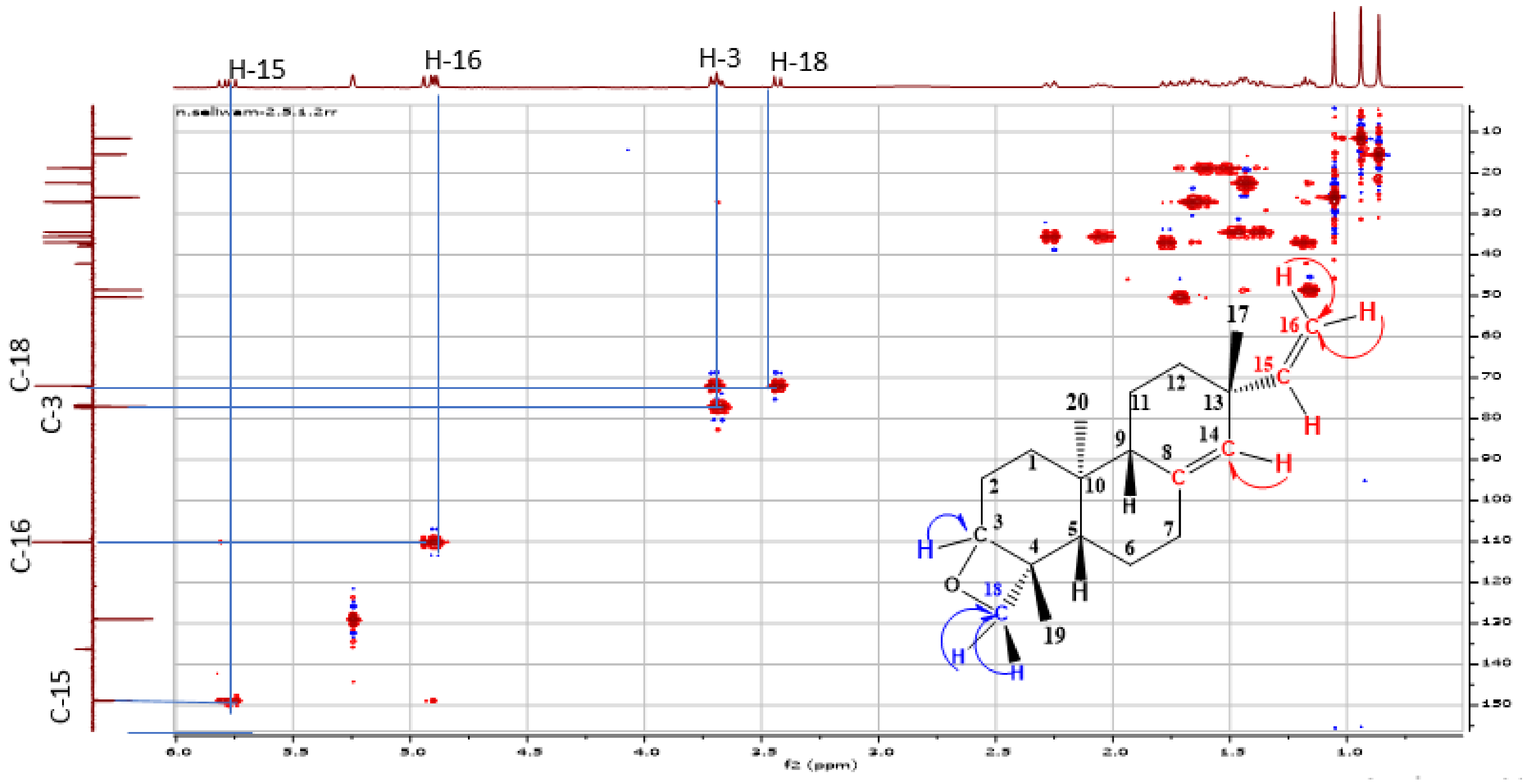

Figure S6. HSQC spectrum of 18-epoxy-pimara- 8(14),15-diene in $CDCl_3$.

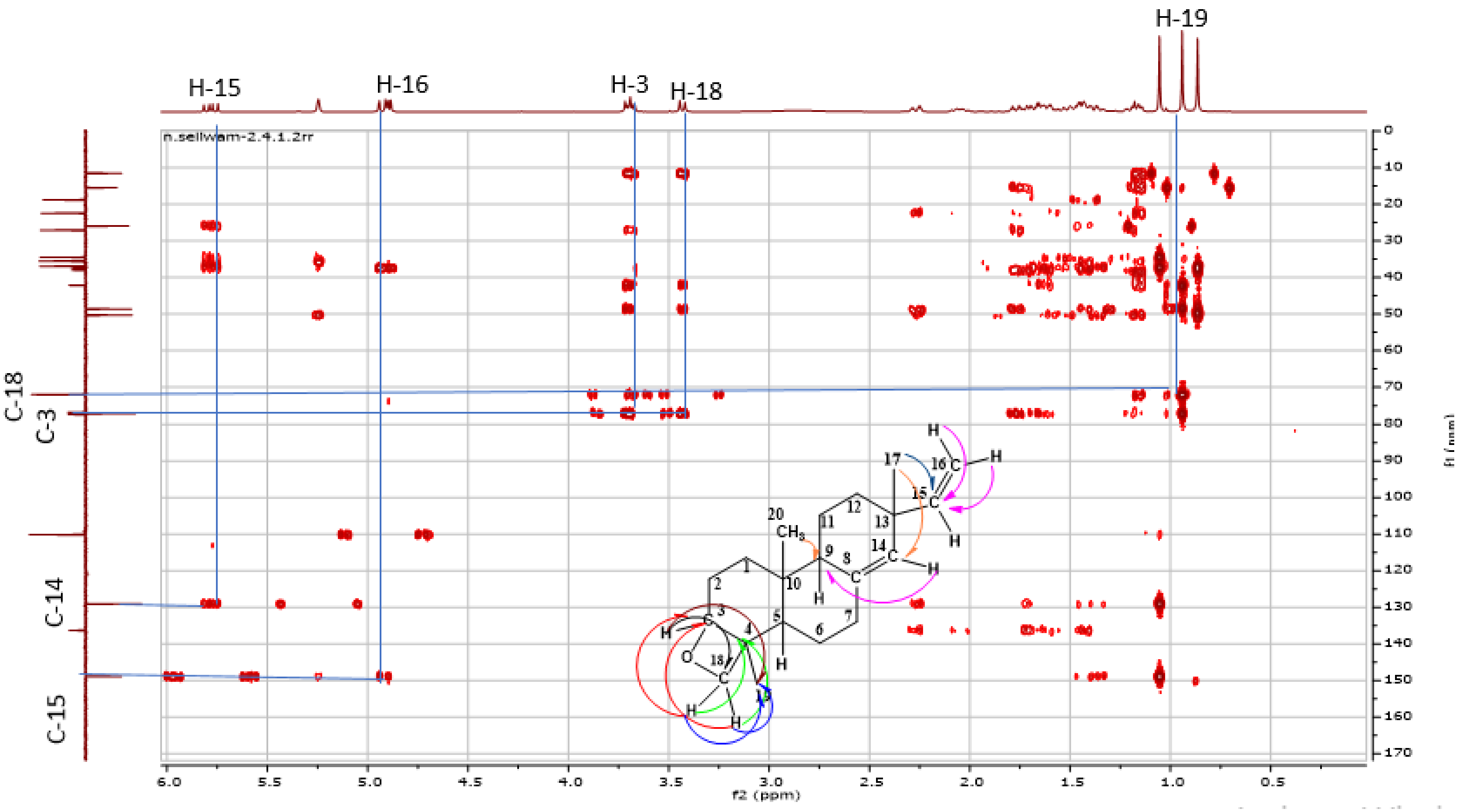

Figure S7. HMBC spectrum of 18-epoxy-pimara- 8(14),15-diene in $CDCl_3$.

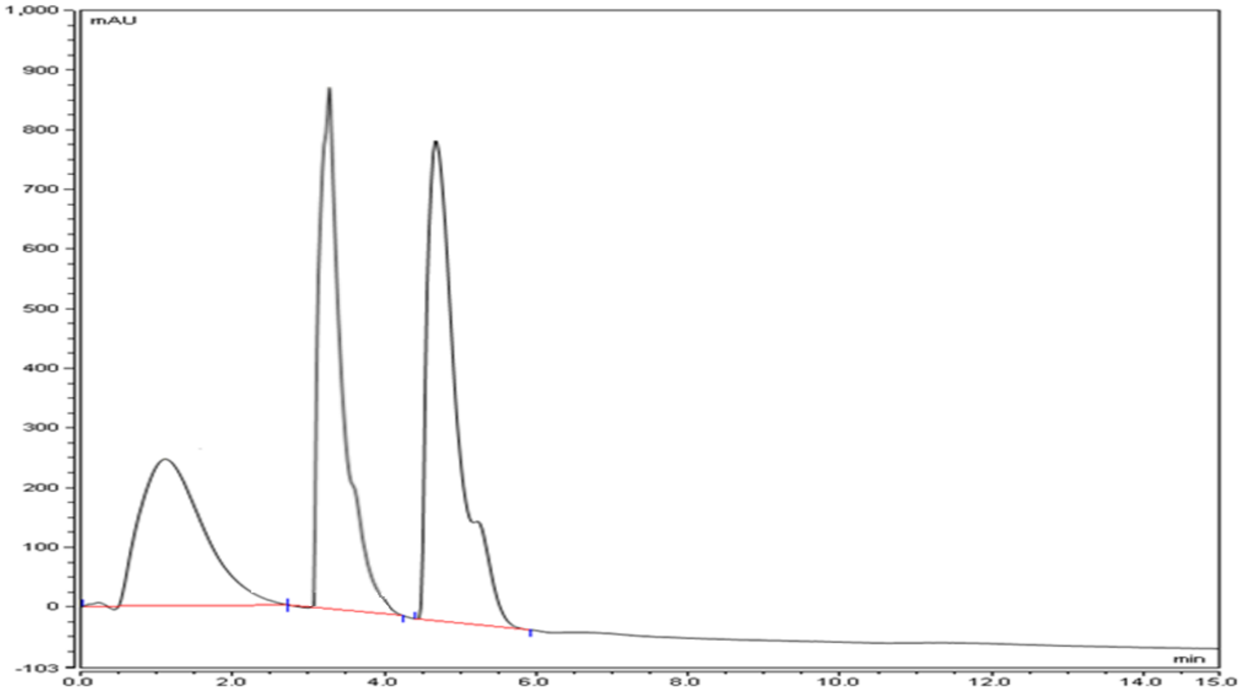

Figure S8. UPLC-PDA chromatogram of *B. grandiflorum n*-hexane fraction.

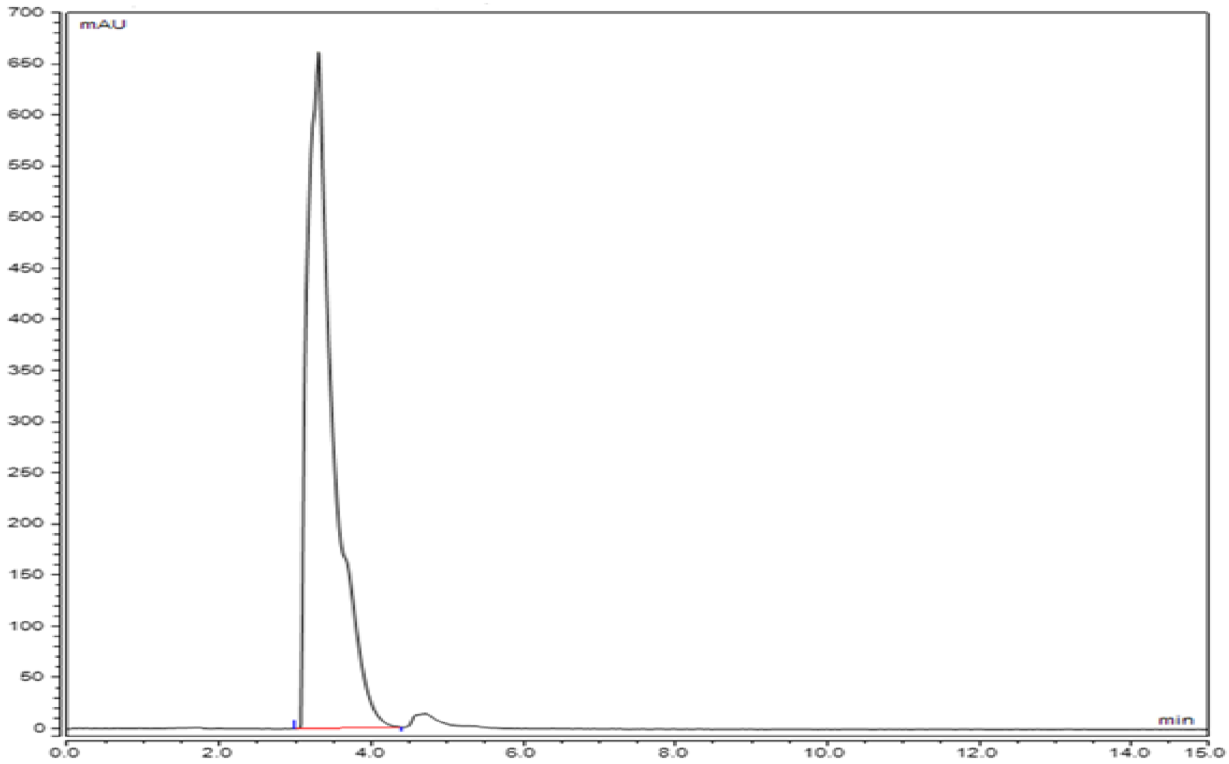

Figure S9. UPLC-PDA chromatogram of 18-epoxy-pimara- 8(14),15-diene.

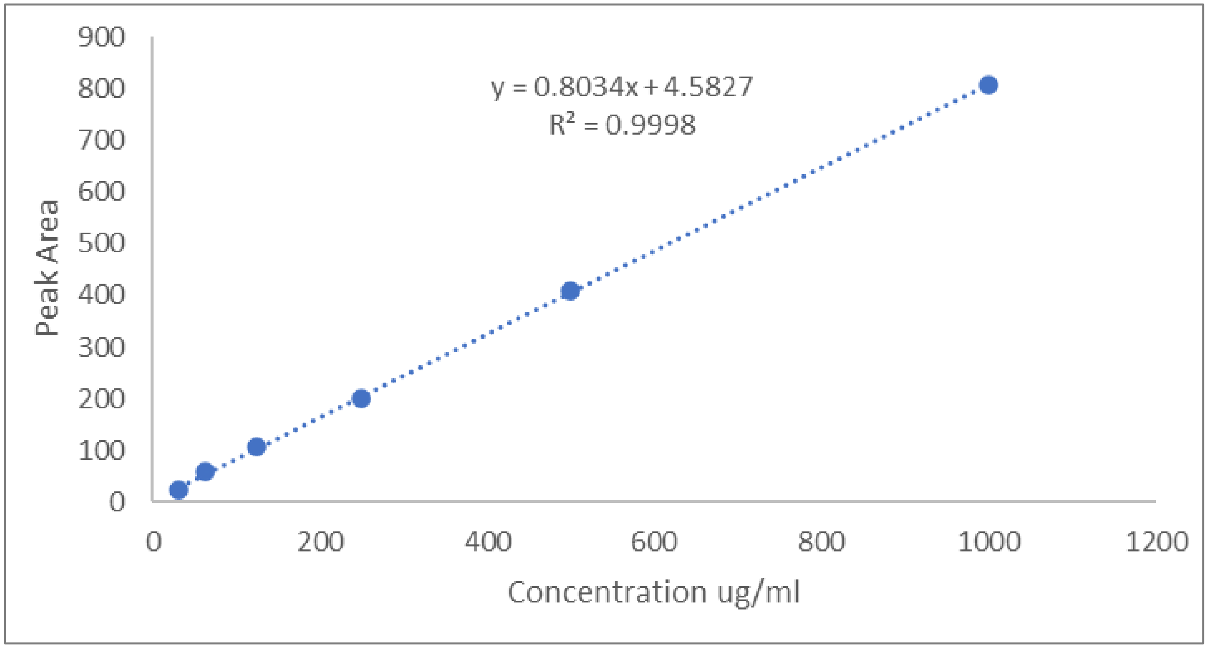

Figure S10. Calibration curve of 18-epoxy-pimara- 8(14),15-diene.

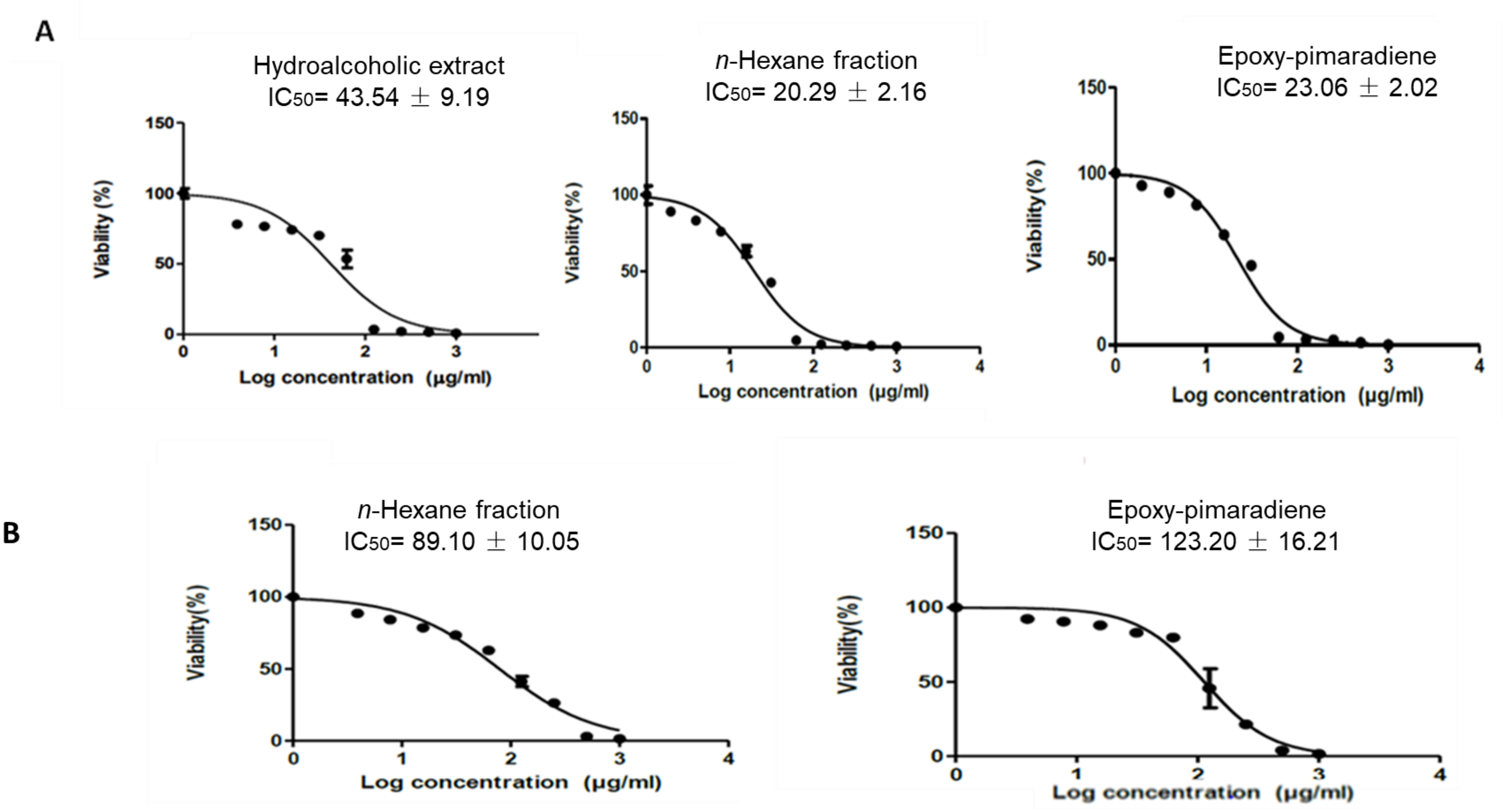

Figure S11 *continued*

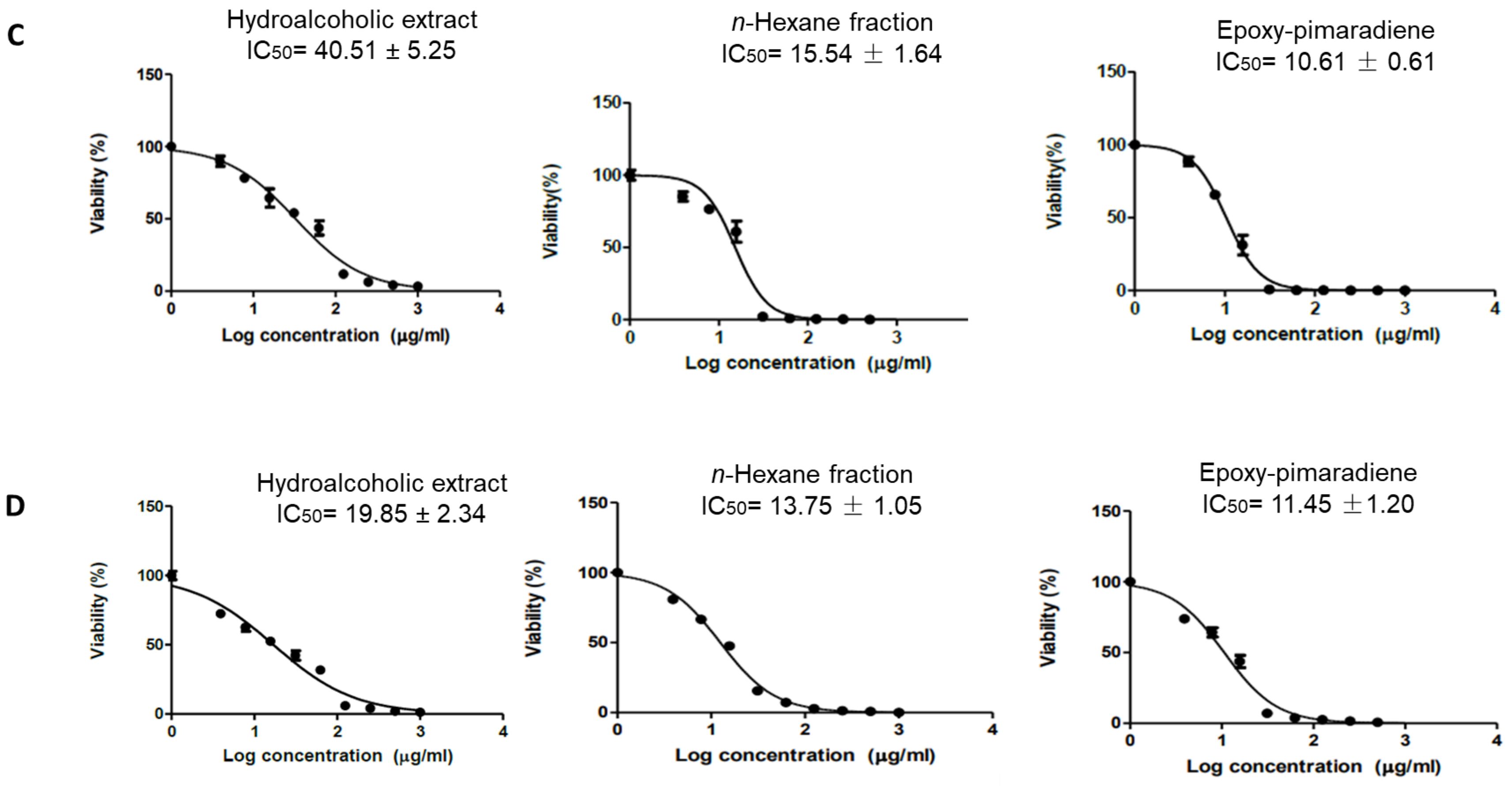

Figure S11 Cytotoxicity of hydroalcoholic extract, *n*-hexane fraction, and compound 2 on **(A)** MCF-7, **(B)** MDA-MB-231, **(C)** HCT-116, and **(D)** HepG2 cell lines.
